# Whole body transcriptomes and new insights into the biology of the tick *Ixodes ricinus*

**DOI:** 10.1186/s13071-018-2932-3

**Published:** 2018-06-26

**Authors:** N. Pierre Charrier, Marjorie Couton, Maarten J. Voordouw, Olivier Rais, Axelle Durand-Hermouet, Caroline Hervet, Olivier Plantard, Claude Rispe

**Affiliations:** 1 BIOEPAR, INRA, Oniris, Université Bretagne Loire, 44307 Nantes, France; 2 Laboratoire d’Ecologie et Evolution des parasites, Institut de Biologie, Université de Neuchâtel, Rue Emile-Argand 11, CH-2000 Neuchâtel, Switzerland

**Keywords:** Transcriptomics, RNA-seq, *Ixodes ricinus*, Expression profiling, Polymorphism

## Abstract

**Background:**

*Ixodes ricinus* is the most important vector of tick-borne diseases in Europe. A better knowledge of its genome and transcriptome is important for developing control strategies. Previous transcriptomic studies of *I. ricinus* have focused on gene expression during the blood meal in specific tissues. To obtain a broader picture of changes in gene expression during the blood meal, our study analysed the transcriptome at the level of the whole body for both nymphal and adult ticks. *Ixodes ricinus* ticks from a highly inbred colony at the University of Neuchâtel were used. We also analysed previously published RNAseq studies to compare the genetic variation between three wild strains and three laboratory strains, including the strain from Neuchâtel.

**Results:**

RNA was extracted from whole tick bodies and the cDNA was sequenced, producing 162,872,698 paired-end reads. Our reference transcriptome contained 179,316 contigs, of which 31% were annotated using Trinotate. Gene expression was compared between ticks that differed by feeding status (unfed *vs* partially fed). We found that blood-feeding in nymphs and female adult ticks increased the expression of cuticle-associated genes. Using a set of 3866 single nucleotide polymorphisms to calculate the heterozygosity, we found that the wild tick populations of *I. ricinus* had much higher levels of heterozygosity than the three laboratory populations.

**Conclusion:**

Using high throughput strand-oriented sequencing for whole ticks in different stages and feeding conditions, we obtained a *de novo* assembly that significantly increased the genomic resources available for *I. ricinus*. Our study illustrates the importance of analysing the transcriptome at the level of the whole body to gain additional insights into how gene expression changes over the life-cycle of an organism. Our comparison of several RNAseq datasets shows the power of transcriptomic data to accurately characterize genetic polymorphism and for comparing different populations or sources of sequencing material.

**Electronic supplementary material:**

The online version of this article (10.1186/s13071-018-2932-3) contains supplementary material, which is available to authorized users.

## Background

Ticks are vectors of numerous pathogenic microorganisms (*Borrelia* spp*.*, *Babesia* spp*.*, tick-borne encephalitis virus, etc.) that cause infectious diseases to both humans and animals [1]. Ticks acquire tick-borne pathogens from infected vertebrate hosts and transmit them to other animals during the blood meal [2]. During blood-feeding, the tick salivary glands secrete a complex cocktail of molecules that allows them to inhibit the different components of the response of the vertebrate host including coagulation, inflammation and immunity [3, 4]. Tick-borne pathogens bind to these tick salivary gland proteins to evade host immunity and enhance their own transmission [5, 6]. Similarly, during acquisition, tick-borne pathogens interact with tick proteins that allow them to persist in the tick midgut [7]. For this reason, anti-tick vaccines have traditionally targeted tick proteins in the salivary glands or midgut in the hope of reducing the efficiency of tick feeding and pathogen transmission [8]. However, a broader knowledge of genes involved in other aspects of the tick life-cycle (e.g. growth and moulting) may lead to alternative control strategies.

*Ixodes ricinus* is one of the most abundant and widespread tick species in Europe where it transmits a number of tick-borne diseases including Lyme borreliosis and tick-borne encephalitis [9, 10]. Hard ticks of the genus *Ixodes* have three motile stages: larva, nymph and adult. The immature stages (larvae and nymphs) take a single blood meal, then moult to the next stage; adult females take a blood meal to produce eggs. There is currently much interest in studying the genome of *I. ricinus* and other tick species in the hope of developing vector control strategies. Recent advances in sequencing technology have made it possible to study large catalogues of gene transcripts (the transcriptome) in individual species. By comparing gene expression between different states (e.g. developmental stage, sex, environmental conditions, etc.), these studies can provide insight into gene function. These RNA-sequencing studies can also provide information on genetic variation within and among populations [11]. To date, several studies have investigated gene expression in *I. ricinus* [12–17]. Most of these studies have focused on gene expression during the blood meal in either the nymphal tick or the adult tick, which is when pathogen transmission occurs. The majority of these studies have investigated gene expression in the tick salivary glands and/or the tick midgut because these tissues are critical for pathogen transmission [12–14, 16]. Taken together, these studies have shown that there are thousands of transcripts that are differentially expressed with respect to the duration of the blood meal, the developmental stage (nymph *versus* adult), the specific tissue (salivary glands *versus* midgut), and other conditions [12–19]. Most of these studies have focused on gene expression during the blood meal in either the nymphal tick or the adult tick, which is when pathogen transmission occurs [14].

The purpose of the present study is to explore new transcriptomic data of *I. ricinus* to improve several aspects of the existing knowledge. First, we wanted to enrich the global catalogue of genes for *I. ricinus*. We used whole tick bodies, which is expected to provide a broader description of the transcriptome compared to the previously published tissue-restricted libraries. We used strand-oriented sequencing, which produces contigs in the direction of transcription for the majority of transcripts. This type of sequencing gives a higher accuracy in the process of gene identification, especially for genes without detectable homology. Secondly, our design included different developmental stages and both sexes in order to capture the highest possible transcriptional diversity. The third innovative aspect of our study was the exploration of high throughput transcriptomic sequencing to detect and compare polymorphism levels among different sources of ticks. Transcriptome sequencing can identify single nucleotide polymorphisms (SNPs) on thousands of coding genes (see [14] for an application for tick data). In the present study, we first studied polymorphism in a highly inbred laboratory strain of *I. ricinus*, which was expected to show low heterozygosity. We then compared the results from our study to the results from previously published RNA-Seq studies that used different sources of *I. ricinus* tick material: (i) wild ticks; (ii) F1 offspring obtained from a mating between two wild ticks; and (iii) a tick cell line. Specifically, we compared levels of polymorphism and heterozygosity between four different sources of *I. ricinus* tick material that were expected to differ with respect to their genetic variability. This information provides a large catalogue of polymorphic sites in expressed regions and provides an important database for future population genetic studies.

## Methods

### Origin of ticks

The *I. ricinus* ticks used in this study came from a laboratory colony reared at the University of Neuchâtel, in Neuchâtel, Switzerland. This colony was initiated in 1978 with a small number of wild ticks collected from a natural population near Neuchâtel and has been maintained as follows. Larval and nymphal ticks are fed on laboratory mice (*Mus musculus*) and adult ticks are fed on rabbits (*Oryctolagus cuniculus*). Completion of the life-cycle of *I. ricinus* (from eggs to eggs) in the laboratory takes about 1 year. Each year, there is at least one cycle of sexual reproduction with a mating population of 40 to 50 adult ticks. There has been no admixture between this tick colony and wild *I. ricinus* ticks for almost 40 years (∼40 generations). The ticks from this colony are therefore expected to be pathogen-free and have reduced genetic diversity due to prolonged inbreeding.

### Preparation of the biological samples

We obtained ticks in five different biological states (sample size in brackets): (i) unfed nymphs (*n* = 60); (ii) partially fed nymphs (*n* = 12); (iii) unfed adult females (*n* = 8); (iv) partially fed adult females (*n* = 4); and (v) unfed adult males (*n* = 12). Sample sizes for cDNA sequencing differed among biological states because the amount of mRNA per individual differed between stages (nymphs *vs* adults) and feeding conditions (unfed *vs* partially fed). Nymphs were fed on mice (*Mus musculus*) and removed after 24 h of attachment while adult females were fed on rabbits (*Oryctolagus cuniculus*) and removed after 48 h of attachment. These feeding durations for both stages (adult ticks feed longer than nymphs) correspond to phase 1 or the slow phase of engorgement following the two phases defined by Lees [20]. During this phase, the nymphs and adults both reach approximately 1/4 of their final engorgement size. Unfed and partially fed ticks were flash-frozen at -80 °C before RNA extractions. To allow statistical comparisons of gene expression levels among the different conditions, three biological replicates were obtained for each of the five combinations of stage and feeding status (a total of 15 samples were prepared).

### Total RNA extraction

Whole tick bodies were ground with a soft plastic pestle in Trizol (Invitrogen, Life Technologies, Carlsbard, CA, USA) on dry ice. RNA was purified as follows: after adding chloroform, the ground material was centrifuged, the aqueous phase was transferred into an RNase-free tube and was topped up with ethanol. RNA was extracted using a NucleoSpin RNA XS column (Macherey-Nagel, Düren, Germany), which included a DNAse treatment. A second DNase treatment (Machery-Nagel) in RNasin (Promega, Madison, USA) was performed to ensure complete degradation of any remaining genomic DNA. The absence of genomic DNA was confirmed by PCR tests that targeted the *18S* ribosomal RNA gene of *I. ricinus*.

### Library preparation and sequencing

The quantity and quality of extracted RNA was evaluated with NanoDrop (Thermo Fisher Scientific, Waltham, USA), Qubit (Invitrogen, CA) and Experion machines (Bio-RAD Laboratories Inc., Hercules, USA). All samples had sufficient quantities, concentrations and qualities of RNA to proceed with library preparation. The library preparation kit was NEBNext® Ultra Directional RNA Library Prep Kit, NEB Art. No E7420. Poly-A selection with a magnetic isolation module was used to target mRNAs, followed by strand-specific cDNA synthesis with an insert size of 150–400 bp, PCR amplification and library purification. Individual tags used for the 15 samples allowed multiplex sequencing. Sequencing was done on one lane of an Illumina Hiseq 2500 machine (v4 chemistry).

### Quality and assembly of reads

To produce the dataset, the raw paired-end reads (2 × 125 bp) were first cleaned. Adapters were clipped and low-quality regions were filtered using Trimmomatic (release 0.36) [21]; only reads with a minimum of 36 high quality-scored contiguous bases were kept. Summary statistics of the sequence quality were checked for each library by visualizing the FastQC report (release 0.11.5) [22].

### Filtering out rRNA reads and *de novo* assembly

To filter out reads corresponding to rRNA gene expression, reads were mapped to a large contig encompassing the *18S*, *28S* and *5.8S* ribosomal genes. This contig was obtained by performing a preliminary *de novo* assembly of publicly available Illumina transcriptomic sequence data for *I. ricinus* as of June 2014 (see Additional file 1: Table S1 for the description of this contig). Reads were mapped to this contig using Bowtie 2 [23]. This contig was expected to be more effective for removing the rRNA reads than the published rRNA sequences of *I. ricinus* because the former contains complete or nearly complete sequences whereas the latter only contains partial sequences. All the reads that did not map to rRNA were assembled with Trinity (release 2014-07-17) [24], using the ’dUTP library preparation’ option to take into account strand-oriented sequencing.

### Assessment of transcriptome completeness

A common test used to assess the “coverage” (or information completeness) of a given sequence dataset is to analyse random samples of reads, and to create a saturation curve describing the relationship between different metrics (numbers of contigs of a specified length, number of matches to a known set of genes, etc.) and the read sample size. Complete datasets have saturation curves that plateau more quickly compared to incomplete datasets. To determine the coverage or completeness of our dataset, we randomly sampled 1, 2, 5, 10, 20, 50, 80, 100, 140 and 160 million reads from our cleaned libraries, as detailed below. Completeness was also estimated by determining the presence of homologs of conserved arthropod genes using the BUSCO approach [25]. BUSCO v1 uses a reference database of 2675 conserved arthropod genes (BUSCO genes) and searches for potential homologs in the database of interest by running BLAST [26] and HMMER [27]. Conserved BUSCO genes are assigned to four classes of genes: (i) missing; (ii) fragmented; (iii) duplicated; and (iv) complete. To determine if the open reading frames (ORFs) were correctly predicted, we checked the strands of the predicted genes within the contigs matching the BUSCOs. To produce the final assembly, we reduced the potential redundancy resulting from the presence of alternative transcripts in the contigs. We clustered similar sequences using cd-hit-est [28] with 98% of identity, retaining the longest transcript of each cluster. Identity parameters were chosen to cluster nearly identical sequences resulting from alternative splicing. We used relatively stringent parameters for clustering: the local alignment had to comprise more than 50% of the longest alignment and more than 80% of the shortest alignment. To assess the loss of information produced by the clustering, we checked read recruitment in our final set of contigs by mapping with Bowtie 2 [23].

### Gene prediction and annotation

The prediction of coding sequences (> 100 amino acids) was performed using TransDecoder, which is part of the Trinity software [24]. The TransDecoder options were set to account for the strand orientation of the sequencing (i.e. ORFs were searched only on the forward strand). Finally, annotations from comparison with public databases were used to filter multiple ORF predictions by transcripts (see below). Following the pipeline recommendation of Trinotate (release 2.0.2) [29], both contigs and predicted peptides were compared by blastx+ and blastp+ (release 2.2.29) [26] to releases of Swissprot and Uniref90 (available at https://data.broadinstitute.org/Trinity/__deprecated_trinotate_resources/Trinotate_v2.0_RESOURCES/ v2.0 RESOURCES/). Protein domains were identified using HMMER (release 3.0 from March 2010) [27] with PFAM-A [30], signal peptides with SignalP [31], and transmembrane domains with TMHMM [32]. We tagged ribosomal RNAs using RNAmmer [33]. All these layers of annotation were combined by Trinotate to assign gene ontology (GO) information to each contig. In the case of multiple ORF predictions for a contig, if one ORF was similar to a known protein while the others was not, only the former ORF was retained. In other cases (several ORFs with similarity to known proteins, or several ORFs with no similarity to known proteins), the different ORFs were retained. As we were particularly interested in cuticular proteins, all peptides that contained the chitin-binding domain (PF00379) were classified using the CutProtFam-Pred webserver [34].

### Comparison of the completeness of our assembly relative to other assembled transcriptomes

In recent years, several research groups have produced RNAseq datasets for *I. ricinus*. For most of these projects, the *de novo* assemblies (or sets of predicted genes derived from these assemblies) have been published in the Transcriptomes Shotgun Assembly (TSA) division of GenBank. Using statistics provided by BUSCO [25], we compared the completeness of our final assembly to that of six different assemblies/gene sets, which were obtained from six different RNAseq projects (see Additional file 1: Table S2; TSA accessions: GADI01, GANP01, GBIH01, GCJO01, GEFM01 and GEGO01). In addition, to determine the relative contribution of each of the different datasets (TSA and or own CDS prediction) to the complete gene collection, we analysed the clustering of all CDSs using cd-hit-est with default parameters [28] -for GCJO01, which corresponded to contigs, we predicted CDSs by using Transdecoder [28].

### Differential expression and GO enrichment

In addition to the reconstruction of transcript sequences, RNA sequencing also allows the user to quantify transcript expression by counting the number of sequenced reads that map to a given transcript. Paired reads for each library were pseudo-aligned on the Transcriptome de Bruijn Graph (T-DBG) using Kallisto [35]. We chose this method, based on a k-mer approach, because it is much faster while providing the same accuracy as the best mapping approaches [35]. This method produced raw counts and normalized count statistics (TPM, or transcripts per million reads) for each assembled contig. These counts allowed us to test for differential expression between conditions. To assess feeding-related changes in gene expression, we compared partially fed ticks *versus* unfed ticks. For this, we performed a comparison between two ensembles of libraries, respectively D/E/F/M/N/O and A/B/C/G/H/J/K/L. Differential expression analyses were performed with the R package *DESeq2* [36] using the raw counts from Kallisto, each library been taken as an independent replicate. To describe the relevant biological changes between conditions, we used predictions produced by gene ontology (GO) term annotation, which included: “molecular function”, “biological process” and “cellular localization” [37]. GO terms were compared between transcripts that were not differentially expressed between conditions (unbiased transcripts) *versus* transcripts that were differentially expressed between conditions. We defined unbiased transcripts as contigs with no significant change in expression between conditions (fold change less than 2 and adjusted *P*-value higher than 0.05). Enrichment analysis was performed using the *elim* method using Kolmogorov-Smirnov tests developed and implemented by Alexa [38] in the R package *TopGO*. As suggested by this author, multiple testing was taken into consideration by using the false discovery rate (FDR) on the enrichment test *P*-values. The resulting GO enrichments were analyzed using the R package *GOprofiles* [39], which provides visualization tools. GO enrichment comparisons are by definition limited to contigs with assigned GOs, whereas many more contigs can show significant changes in expression between conditions. A substantial number of contigs had no assigned GOs but did have other annotations, such as domains identified through the PFAM analysis. We therefore used a text mining analysis of the PFAM domains to further compare changes in gene expression between the different conditions (unfed/fed, nymph/adult, male/female). This approach allowed us to distinguish the most common terms associated within a text. We therefore extracted the PFAM terms associated with contigs over-expressed in each of the conditions defined above (fed or unfed) and treated them as a single text for each category. To prevent over-representation of transcripts with many PFAM domains, only the first ten PFAM terms with the lowest e-value were retained for a transcript and over-expressed contigs were defined as contigs with a fold change larger than 4 and a significant *P*-value (*P* < 0.05) in the DESeq2 analysis. PFAM descriptions were edited to remove the less informative terms (e.g. “protein”, “domain”, “motif”). We then used an in-house R script to draw clouds of words with word sizes proportional to their frequency in the text. This approach is complementary to the GO-enrichment tests, as it helps to visualize major shifts in expression among conditions.

### Summary of results by peptide predictions and by contigs

An annotation file with tab-separated values was produced, providing all the information from the Trinotate report, raw counts from Kallisto, log fold changes and *P*-values for differential expression, and BUSCO information. This report contains one line per peptide prediction and was deposited with a DOI on the Zenodo platform (see the section “Availability of data and materials” below).

### Polymorphisms

Polymorphism was surveyed in the reads produced through our project (corresponding to an inbred line, here after referred to as NEU) but also for data from five other RNAseq projects of *I. ricinus* publically available in GenBank. Three published datasets used wild tick populations from Sénart in France (SEN) [16], from the Czech Republic (CZ-W) [13], and a mixture of wild tick populations (LUX) that was provided by Charles River Laboratory [17]. The other two datasets were based on F1 full sibs from a cross between wild ticks from the Czech Republic (CZ-F1) [19], and a tick cell line (CL) deposited by the Broad Institute under BioProject accession numbers PRJNA238785-88. More details on those 5 datasets are given in Additional file 1: Table S3. Reads sequences are available at the NCBI Sequence Reads Archive (SRA) and organized by BioProject. After downloading reads from the SRA archives, the reads were cleaned using Trimmomatic (with the same parameters as above). As estimates of polymorphism and heterozygosity depend on sample size, we standardized the sample size for each dataset by randomly sampling 30 million reads from each SRA. The combined datasets were analysed to detect single nucleotide polymorphisms (SNPs). SNPs were predicted with a “direct from the reads” approach, using KisSplice (release 2.4.0) [40], a software that identifies variations by detecting “bubbles” in the De Bruijn graph. SNPs were mapped on our final set of contigs using Blat (version 36) [41]. A report assessing various parameters for each SNP (location, reliability, etc.) was provided by Kiss2refTranscriptome [42]. To minimize false positives, we retained only SNPs that respected the following criteria: (i) SNPs had to be covered by at least ten reads in each dataset; and (ii) SNPs needed to be uniquely mapped (e.g. mapping to a single component). Using this restricted set of SNPs, we used variant counts produced by KisSplice to calculate allele frequencies at each polymorphic site, for each of the 6 RNAseq datasets. We estimated heterozygosity (using the formula He = 2pq, where p and q are the frequency of each variant) for each SNP position, and for each dataset.

## Results

### Reads and assembly statistics

We obtained a total of 210,229,106 paired strand-oriented reads. After filtering out the poor-quality reads and orphan reads (13,611,318 reads) and after excluding the reads assigned to rRNA (33,745,090 reads), the final set contained 162,872,698 trimmed, good-quality reads (see Table 1). For the fifteen libraries, the mean number of reads was 10,858,180 reads per library (range 7,628,548–15,814,366). Trinity produced an initial assembly set of 427,491 contigs, of which half had a length between 200–300 bp. As these small contigs are expected to be mostly represent gene fragments or untranslated region (UTR) sequences, we discarded them and only considered contigs above 300 bp. After reduction of redundancy [28], the assembly contained a total of 192,050 contigs. For the 15 different tick libraries, 88% and 91% of the reads mapped back to contigs (see Table 1). This good recruitment rate suggests that (i) the Trinity-produced assembly managed to capture most of the information contained by the reads, and (ii) little information was lost after eliminating the smaller class of contigs (< 300 bp). As an internal test of completeness, we analyzed the numbers of contigs of different sizes that resulted from the assemblies of random subsets of reads of increasing sample size. There was no clear saturation when considering all contigs, as the number of contigs was still rising (with only a moderate decrease of the slope) for even the largest read sample sizes (see Additional file 1: Figure S1). However, when only considering contigs above a certain size, the number of contigs clearly tended to plateau; this plateau can already be seen for contig size > 300 bp, whereas the plateau was marked for contigs > 1000 bp. This saturation effect was also shown with the BUSCO approach where the numbers of conserved arthropod genes plateaued at a sample size of 100 million reads (see Additional file 1: Figure S2). Overall, this result suggests that our complete set of reads tended to saturate the information on the mid-size to large transcripts, indicating that the coverage of our *I. ricinus* transcriptome was good (when considering all together the 5 combinations of stage, sex, and feeding conditions in our study).

**Table 1:** Description of the 15 libraries. The 15 libraries refer to the 5 combinations of stage, sex, and feeding condition for *I. ricinus* ticks, each replicated 3 times. The different columns refer to: library identification code, stage, sex, feeding condition, number of cleaned quality reads (Reads), percentage of reads that mapped back to the final set of transcripts with Bowtie 2 (Recruitment) and number of counting events observed by Kallisto divided by the number of paired reads (Kallisto)

Library	Stage	Sex^a^	Condition	Reads	Recruitment (% reads)	Kallisto (% reads)
A	Nymph	Unknown	Unfed	13,839,882	88.1	86.5
B	Nymph	Unknown	Unfed	9,158,226	86.9	85.7
C	Nymph	Unknown	Unfed	13,233,880	88.4	86.5
D	Nymph	Unknown	Fed	12,665,880	89.5	87.8
E	Nymph	Unknown	Fed	8,230,200	91.4	89.9
F	Nymph	Unknown	Fed	7,762,182	89.5	88.0
G	Adult	Male	Unfed	8,191,900	91.5	89.7
H	Adult	Male	Unfed	9,360,492	90.7	89.0
I	Adult	Male	Unfed	11,228,194	91.6	90.7
J	Adult	Female	Unfed	11,604,308	91.6	89.6
K	Adult	Female	Unfed	15,814,366	91.2	88.8
L	Adult	Female	Unfed	10,277,418	90.9	88.7
M	Adult	Female	Fed	12,813,758	90.5	89.1
N	Adult	Female	Fed	11,063,464	94.7	94.5
O	Adult	Female	Fed	7,628,548	90.6	90.2
Total				162,872,698	90.4	88.9

^a^Sex is unknown for nymphs

### Taxonomic assignation

**Fig. 1:**
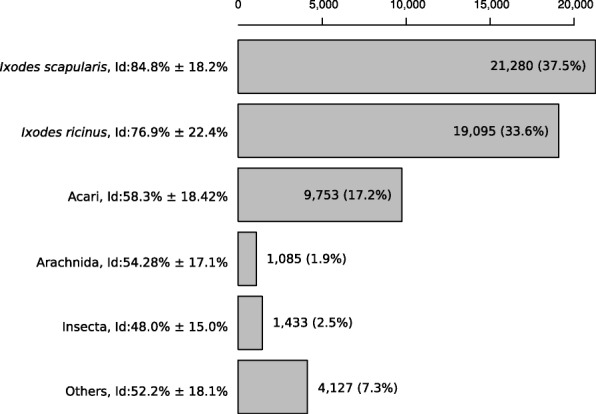
Taxonomic assignment of contigs using Uniref90 best blast hits. The bars correspond to the number of contigs assigned to different taxonomic groups (the percentage of contigs assigned to each taxonomic group is shown in brackets). Best hits to “Acari”, which include ticks and mites are hits to Acari species other than *I. ricinus* and *I. scapularis*. Best hits to “Arachnida”, which also include spiders and scorpions, are hits to Arachnida species other than Acari. The mean percentage of identity at the amino acid level and the standard deviation are shown after the taxon name

We used taxonomic information from the code name of the best hit on the Uniref90 proteins cluster. We found that 6.4% of assembled transcripts (*n* = 12,368) were assigned to fungi (considering a minimum of 50% of protein identity and an E-value lower than 10e-5). Fungal contamination of individual ticks has been observed in the *I. ricinus* colony at the University of Neuchâtel. To facilitate moulting, blood-engorged larvae are placed in tubes with moistened filter paper, which also facilitates the growth of opportunistic fungi, which are the most likely source of the contamination observed in the present study. We counted 887,767 reads that mapped to the fungi-like contigs, which represents only 1.2% of the total number of counting events by Kallisto, suggesting that contamination was modest. Statistics on the abundance of these fungi-like transcripts for each library are presented in Additional file 1: Figure S3, which showed that this contamination was restricted to unfed nymphs. The partially fed nymphs and adults contained blood (and therefore RNA) from mice and rabbits, respectively. After removing the 12,368 fungi-like contigs and 366 mammalian transcripts, the final assembly contained 179,316 contigs (Table 2). A taxonomic assignation based on the best blastx hit on Uniref90 was obtained for 56,773 contigs; of these, 37.5% and 33.6% were assigned to *Ixodes scapularis* and *I. ricinus*, respectively (Fig. 1). Another 17.2% of the contigs had matches to “Acari” (i.e. tick and mite species other than *I. scapularis* and *I. ricinus*) and 2.5% had matches to “Insecta” (*n* = 1433 contigs). The insect species with the most abundant hits were: pea aphid *Acyrthosiphon pisum* (*n* = 590 contigs), termite *Zootermopsis* (*n* = 145), clonal raider ant *Ooceraea biroi* (*n* = 69), Asian long-horned beetle *Anoplophora glabripennis* (*n* = 66), and kissing bug *Rhodnius prolixus* (*n* = 6267). As expected, the mean identity of the hits reflected phylogenetic distance so that identity was highest for hits corresponding to *Ixodes* species, and lower for distant taxonomic groups. However, this expected pattern was reversed at the finest taxonomic level (genus *Ixodes*), where the mean distance to *I. scapularis* hits was lower than the mean distance to *I. ricinus* hits. We explored the distribution of % identities at the amino acid level (see Additional file 1: Figure S4) for best hits to *I. ricinus* or *I. scapularis*. For both species, the highest peak of genes had a very high percentage of identity (> 95%). This peak probably corresponds to the orthologous genes found in both *I. scapularis* and *I. ricinus*. However, the distribution of identities for *I. ricinus* shows a secondary peak of hits with a low identity (∼45%). These low identity hits cannot correspond to the same gene, but probably represent distant paralogs of genes, or genes having a similar protein domain. The presence of this secondary peak decreases the mean identity of hits to *I. ricinus* and explains why the mean identity of our dataset is lower for *I. ricinus* than *I. scapularis*.

**Table 2:** Statistics of assembly for the final set of contigs: total size of contigs, shortest and largest contigs, mean and median contig size and the N50 contig length are expressed in base pairs

Statistic	Assembly
Number of contigs	179,316
Total size of contigs (bp)	130,913,381
Shortest contig (bp)	301
Longest contig (bp)	20,233
Number of contigs > 0.5 kbp	79,235
Number of contigs > 1 kbp	29,911
Number of contigs > 10 kbp	19
Mean contig size (bp)	730
Median contig size (bp)	461
N50 contig size (bp)	875

### Annotation

Overall, 56,809 contigs (31.7% of the total) had a significant similarity with known proteins present in Uniref90 or Swissprot (Table 3). The percentage of contigs with a match strongly increased with contig size. For example, the percentage of contigs with a match was as high as 70.8% for contigs longer than 1 Kb. TransDecoder predicted 57,257 peptides, of which 35.6% were predicted as complete. In total, 13,308 GO terms were extracted from 26,702 contigs (14.9% of all contigs). Transmembrane domains and peptide signals were found for 8869 peptides and 3485 peptides, respectively. In addition, 22,082 contigs (12.3%) had a detected PFAM domain. Some contigs had only a GO match or only a PFAM assignation, so these data were complementary.

**Table 3:** Summary of annotation statistics for the final set of contigs. For each class of contig length the following data are given: number of contigs, number of contigs with a significant hit on Swissprot or Uniref90, number of peptides predicted by TransDecoder and number of contigs for which gene ontology terms could be extracted

Class	No. of contigs	No. of annotated (%)^a^	No of peptides (%)^b^	TGO^c^
301-500	100,081	19,017 (19.00)	16,289 (16.28)	5247 (5.24)
501-1000	49,324	16,604 (33.66)	16,295 (33.04)	6753 (13.69)
1001-1500	13,214	7658 (57.95)	8406 (63.61)	4442 (33.62)
1501-2000	6446	4641 (72.00)	5368 (83.28)	3185 (49.41)
2001-2500	3712	3033 (81.71)	3555 (95.77)	2271 (61.18)
2501-3000	2289	1930 (84.32)	2387 (104.28)	1500 (65.53)
3001-4000	2394	2182 (91.14)	2737 (114.33)	1779 (74.31)
4001,5000	1025	956 (93.27)	1179 (115.02)	814 (79.41)
5001-7500	716	676 (94.41)	882 (123.18)	602 (84.08)
7501-10000	96	93 (96.88)	132 (137.50)	91 (94.79)
10001-100000	19	19 (100.00)	27 (142.11)	18 (94.74)
Total	179,316	56,809 (31.68)	57,257 (31.93)	26,702 (14.89)

^a^Number of contigs with a significant hit on Swissprot or Uniref90

^b^Number of peptides predicted by TransDecoder

^c^Number of contigs for which gene ontology terms could be extracted

### Completeness

**Fig. 2:**
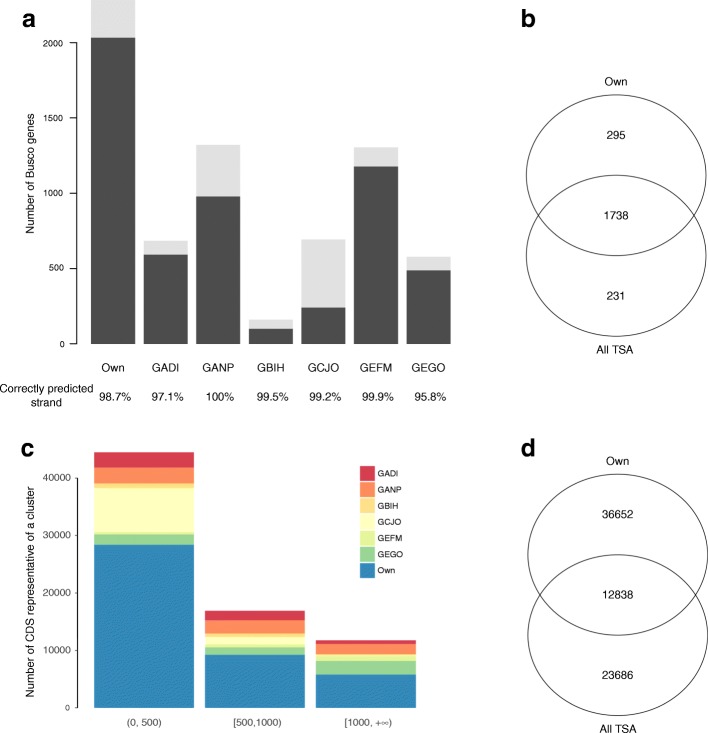
Completeness of our *I. ricinus* transcriptome compared to Transcriptome Shotgun Assembly (TSA) datasets. Completeness was assessed through a comparison with the BUSCO database and by a direct comparison among CDSs from all datasets (cd-hit-est). **a** Number of BUSCO genes found in our assembly (“Own”) and the six TSA projects. The percentage of complete BUSCO genes predicted on the correct strand are shown below each bar. **b** Venn diagram showing the overlap between the number of complete BUSCO genes found in our assembly and in the combined TSA assemblies. **c** Relative contribution of each dataset (different TSA and our assembly) to the combined clustering for three classes of size of contigs. **d** Venn diagram showing how many CDSs are unique and shared between our assembly and published TSA datasets

Of the 2675 conserved arthropod genes in the BUSCO database, our assembly contained 2033 complete genes (completeness of 76%) and 250 partial genes (extended completeness of 85.3%). Previously published assemblies or collections of predicted genes (TSA archives) for *I. ricinus* all produced lower percentages of complete conserved genes (Fig. 2a). A comparison of the overlap between our new dataset and the combined TSA datasets found that our assembly contains 295 BUSCO genes (11%) not present in any of these TSA datasets, whereas the combined TSA datasets contained 231 BUSCO genes not present in our assembly (Fig. 2b). We obtained a similar percentage of BUSCO contigs with correctly predicted strands (98.7%) compared to the combined TSA datasets (mean of 98.6%, minimum of 95.8% for GEGO, maximum of 100% for GANP). We then performed a direct comparison between all the CDSs from our assembly and from the TSA datasets, with a clustering approach. We showed that our assembly permitted to identify 36,652 new CDSs which were not present in any of the published dataset (Fig. 2d). On the other hand, TSAs also contained specific CDSs not found in our study (*n* = 23,686). For CDSs which were represented both in our assembly and TSA datasets, we found that our assembly often produced the longest and most complete CDS. The relative contribution of each dataset and for different length classes is displayed in Fig. 2c: it shows that our assembly contributed to enriching the collection both for small and large CDSs.

### Differential expression

**Fig. 3:**
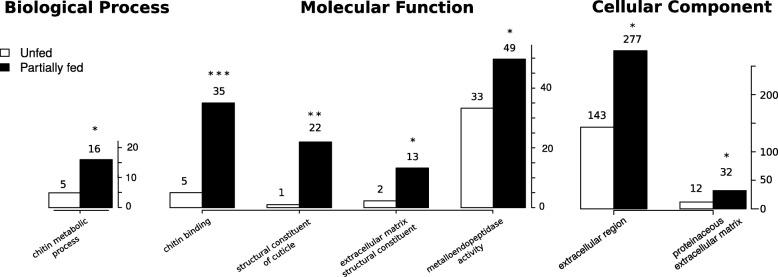
Abundance of significantly enriched GO terms in partially fed ticks. For comparison, the abundance of the same terms for differentially expressed transcripts in both conditions (unfed and partially fed ticks) are also represented. GO enrichment for the comparison between unfed and partially fed ticks. The X-axis shows the top enriched GO terms for the three categories: Biological Process, Molecular Function and Cellular Component. The Y-axis shows the number of GO terms found in differentially expressed transcripts for unfed and partially fed ticks (see Additional file 1: Tables S4-S9 for detailed GO results). Significance of the Kolmogorov-Smirnoff enrichment test corrected by Benjamini & Hochberg: *P < 0.05, ** *P* < 0.0005, *** *P* < 0.000005

**Fig. 4:**
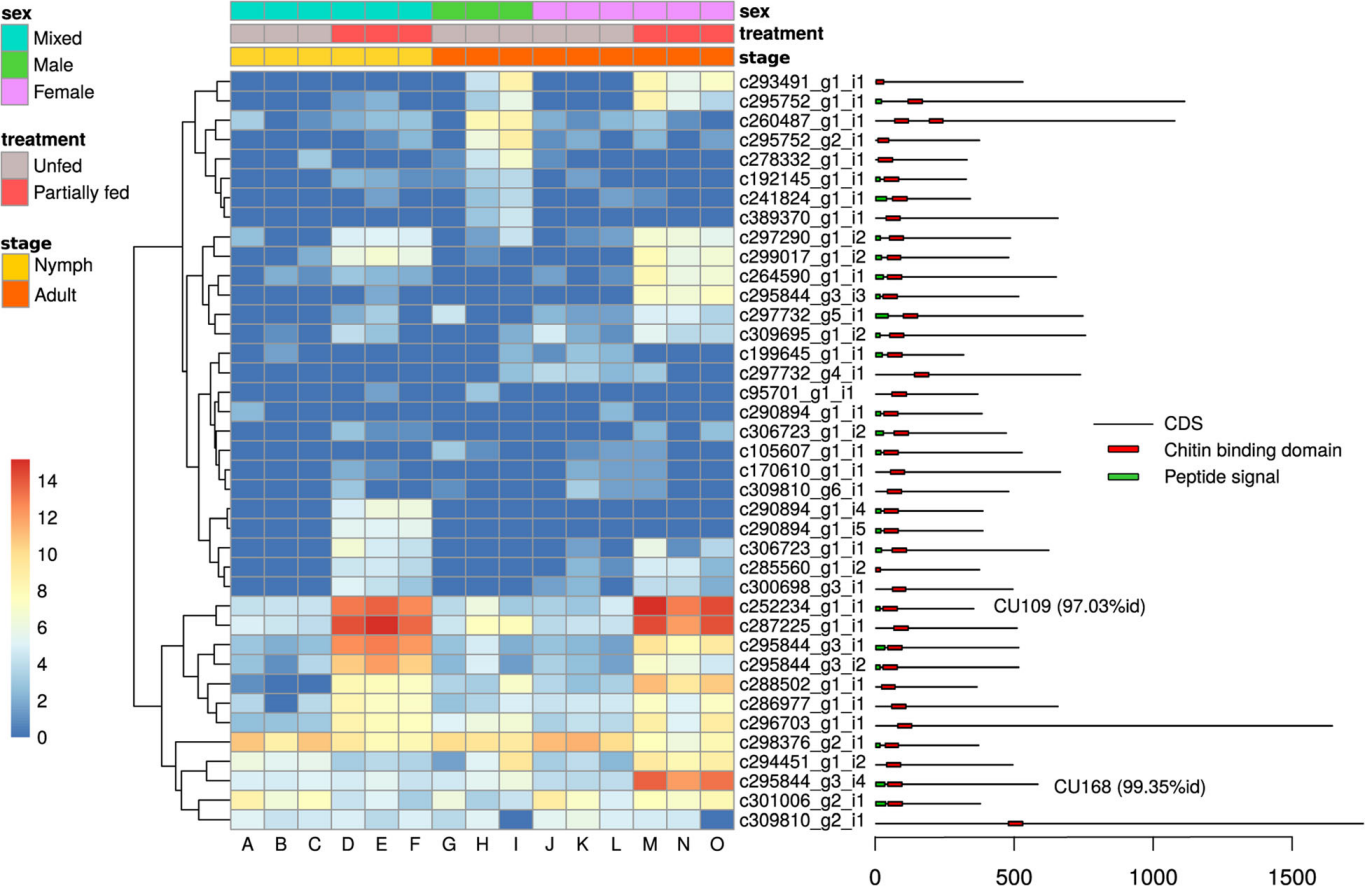
Condition-dependent expression of genes that contain a chitin-binding domain (PFAM domain PF00379). The first three rows above the heat map indicate the sex, seeding treatment and stage condition of the *I. ricinus* ticks in each of the 15 libraries (columns A to O), according to a color legend detailed on the left. Each row of the heat map corresponds to a different gene (contig). Colors of the cells represent the level of expression (log(“raw-count”+1)) of the gene in each library (counted by Kallisto). The legend on the right shows the position of the signal peptide domain and of the chitin-binding domain on the coding sequence of the transcript (CDS). Best-hit UNIPROT names are indicated for genes with more than 90% protein identity

**Fig. 5:**
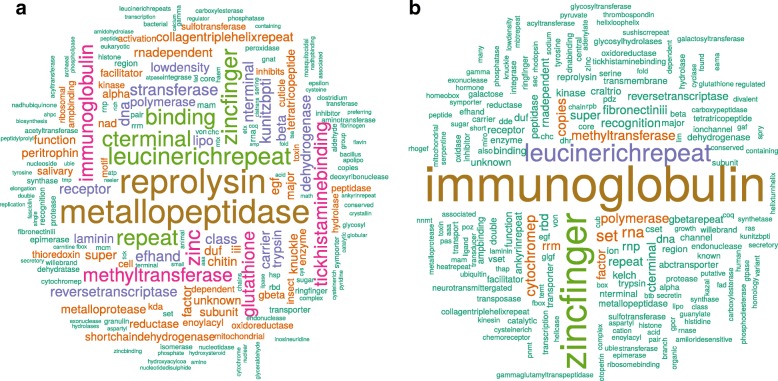
Relative abundance of terms in the identified PFAM domains of transcripts over-expressed in unfed and partially fed ticks. Over-expressed transcripts were defined as transcripts with a fold-change > 4 and a *P*-value < 0.05 (DESeq2 analysis). Terms were extracted from the PFAM annotations retaining the first 10 domains (with the lowest e-value). Terms or characters that were not informative (e.g. “protein”, “domain”, “motif”, “dots”, etc.) were trimmed. Most abundant terms in transcripts upregulated in partially fed ticks (**a**) or upregulated in unfed ticks (**b**)

When comparing the 200 most expressed transcripts, the “I” library of unfed adult males showed a strong deviation from all other libraries, including the two other replicate libraries of unfed adult males (libraries “G” and “H”) (see Additional file 1: Figure S5). The “I” library also contained a particularly high percentage of rRNA reads (> 60%) suggesting that this sample was of lower quality. For this reason, we decided to exclude the “I” library from the analyses of differential gene expression. Comparing the levels of gene expression among libraries (for all transcripts), we found that libraries clustered by condition for unfed nymphs, fed nymphs, and unfed females (Additional file 1: Figure S6). Such clustering was not observed for the unfed males, whereas only two of the three samples clustered together for the fed females. The number of unbiased transcripts was 39,719. We found that 11,322 transcripts (6.3%) were differentially up- or downregulated between the unfed condition and the partially fed condition (adjusted *P*-value < 0.05). Using the GO terms associated with DE transcripts between unfed and partially fed ticks, we found a significant enrichment (FDR < 0.05) for 4 molecular function (MF) terms (Additional file 1: Table S4), 41 biological process (BP) terms (Additional file 1: Table S5), and 6 cellular component (CC) terms (Additional file 1: Table S6). We represent top-significantly enriched GO terms (using up and down DE transcripts) between partially fed and unfed ticks without distinction of stage and sex (Fig. 3). The comparison between unfed and partially fed ticks found a significant enrichment of terms associated with cuticle production (Fig. 3); specifically, the expression of cuticle-associated genes was significantly higher in partially fed ticks than unfed ticks. Out of 39 transcripts containing a chitin-binding PFAM domain (PF00379), one contained two PF00379 domains and 22 contained a signal peptide. All these transcripts were classified as members of group 2 using the CutProFam-Pred webserver. Expression levels in each library of cuticle-related transcripts are shown in a heatmap (Fig. 4). Of the three most expressed cuticular transcripts, two transcripts shared a high identity with the proteins described by Andersen & Roepstorff [43]. The c252234_g1_i1 transcript in our study had 97.03% identity with the Ir-ACP10.9 protein (belonging to the CU109 cluster of Uniprot) [43]. This transcript was found to be highly over-expressed in partially fed ticks (588-fold change between unfed and partially fed ticks; *P*-value < 0.0001). Another transcript (c295844_g3_i4) that was highly over-expressed in partially fed adult females had 99.35% of identity with the Ir-ACP16.8 protein (belonging to the CU168 cluster of Uniprot) described in the same study [43]. The next and last two GO terms for Molecular Function showing significant enrichment for partially fed ticks were “extracellular matrix structural constituent” (which may also be related with constituents of the cuticle), and “metalloendopeptidase activity”. As we discuss below, previous studies of expression several metalloproteases showed strong fold-changes in expression at different time-points of the blood meal [14]. Text mining of the identified domains in the differentially expressed transcripts provided additional insight on the function associated with each of the conditions (see Fig. 5). For the genes that were over-expressed in partially fed ticks compared to unfed ticks (Fig. 5a), we highlight the abundance of the following terms: reprolysin and metallopeptidase and Kunitz-BPTI (these three terms were often associated in the same transcripts) as well as tick-histamine-binding, immunoglobulin and chitin. For the genes over-expressed in unfed ticks (Fig. 5b), the most common terms were immunoglobulin, zinc-finger and leucine-rich-repeat. Actually, some of the most highly DE genes in unfed ticks had several IgC domains as is the case for a homolog of the gene Turtle. However, this trend was not caused by just one or a few transcripts, but by several genes with a similar structure (probably a gene family).

### Polymorphism

**Fig. 6:**
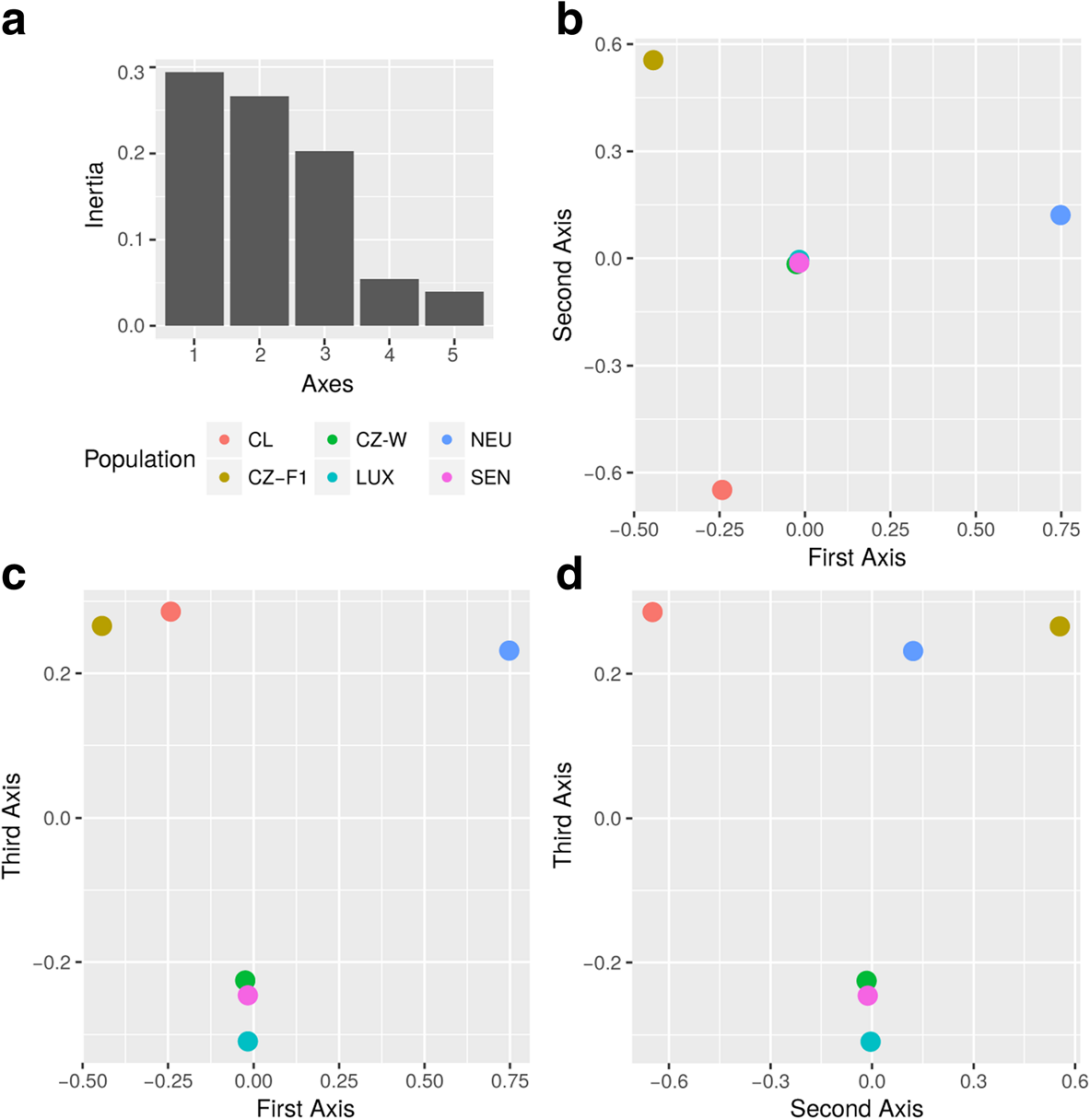
Results of the factorial component analysis (FCA). The FCA explored how six sources of material (from different RNAseq datasets) of *I. ricinus* differ based on the heterozygosity of single nucleotide polymorphism (SNP) loci. The different sources are: wild ticks from the Czech Republic (CZ-W); wild ticks from a commercial strain (LUX); wild ticks from Sénart, France (SEN); a cell line (CL); a first generation of full sibs (CZ-F1); and a laboratory strain (NEU). The variables correspond to heterozygosity at the 3866 selected SNPs. **a** The eigenvalue (percentages of variation or inertia) explained by each eigenvector (axes). **b** The 6 sources on the plane formed by the first two axes. **c** The plane formed by axes 1 and 3. **d** The plane formed by axes 2 and 3

Starting from the 368,367 SNPs initially detected by KisSplice using the default parameters, we applied more stringent criteria to increase the specificity: only 8955 SNPs were supported by at least 10 reads in each strain. We further filtered out all SNPs not assigned unambiguously to a single component of the T-DBG, resulting in a final set of 3866 SNPs. For this small but very robust set of polymorphic sites, the minor allele frequency (MAF) and heterozygosity were computed. A factorial component analysis (FCA) showed that the three wild strains of *I. ricinus* (SEN, CZ-W and LUX) were grouped very closely together (Fig. 6). These three wild tick strains had very similar levels of heterozygosity across loci on the three plane representations (Fig. 6b: plane of axis 1 *vs* 2, Fig. 6c: plane of axis 1 *vs* 3, and Fig. 6d: plane of axis 2 *vs* 3 of the FCA). By contrast, the laboratory strains (CZ-F1, NEU and CL) were distant from the central cluster of wild strains in the plane of axis 1 *vs* 2 space (Fig. 6b) and were also distant from each other. These laboratory strains formed a separate group supported by the third axis (see plane space representation of corresponding to plane of axis 1 *vs* 3 in Fig. 6c and plane of axis 2 *vs* 3 in Fig. 6d). Densities of the loci’s heterozygosities for each strain showed similar patterns (see Additional file 1: Figure S7). Again, the three wild strains showed similar distributions of heterozygosity whereas the laboratory strains (CZ-F1 and NEU) showed very similar profiles: a high proportion of sites with very low heterozygosity (fixed or nearly-fixed SNPs) and a low proportion of sites with intermediate levels of heterozygosity. The CL strain showed an even more striking increase in the proportion of fixed sites. All these results showed that the three laboratory strains have less heterozygosity, differ strongly from wild ticks, and differ strongly from each other.

## Discussion

Several recent RNAseq studies on *I. ricinus* have focused on the transcriptomes of specific tissues such as the midgut, salivary glands, haemocytes and ovaries [13–19]. In contrast, we tried to obtain a broader picture of the transcriptome of *I. ricinus* by sequencing and assembling transcripts from whole ticks in different conditions defined by developmental stage, sex and feeding status. First, our *de novo* assembly showed high overall completeness and significantly enlarged the catalogue of known coding sequences for *I. ricinus*. Secondly, we detected blood-feeding-induced changes in gene expression at the level of the whole body. Thirdly, our analysis of polymorphism in the transcriptome sequence data of our study and previously published studies allowed us to compare the levels of genetic diversity between outbred wild strains of *I. ricinus versus* inbred laboratory strains and tick cell lines.

### Genomic resources for transcribed sequences

Using the BUSCO approach, our assembly showed high completeness with metrics that compared very favorably with previously published *de novo* assemblies (TSA contigs). All of these previous assemblies were based on specific tick tissues (midgut, salivary glands, haemocytes, etc.), which probably contain a smaller repertoire of transcripts which could explain their lower level of completeness. In contrast, we found that 231 BUSCO genes were absent from our assembly but present in the published TSA. By construction, BUSCO genes represent conserved genes found in most arthropods, so this approach does not bring *a priori* much insight for genes specific to ticks (which could be the most significant to understand tick biology). For a more global and more exact comparison, we also compared protein sequences (CDSs) of all published analyses and those predicted in our study. We found again that large numbers of CDSs were predicted only either in our study or in published datasets. Therefore, each analysis had a relatively large level of specificity, a finding thus similar to that of the BUSCO approach. This suggests that it is important to combine the different sources of data to obtain the most complete description of the transcriptome of the species. When analysing shared CDSs in the different analyses (by a clustering method), we found that our predicted CDSs often provided the longest sequence in each cluster, i.e. the most complete gene sequence. Therefore, our predicted collection of CDSs significantly enriched the extant knowledge for *I. ricinus* genes. To understand the differences in completeness between datasets (and their complementary aspects), we stress that tissue-specific transcriptomes (previous studies) will detect transcripts that may be rare at the level of the whole body (our study), whereas our whole-body transcriptome may have helped to identify transcripts present mostly or only in tissues not already covered by previous studies. A second factor is the strong time-dependence of gene expression during the blood meal as shown in previous studies on *I. ricinus* [14] and *I. scapularis* [44]. Our experimental design had only a single time point for nymphs and adult ticks, which might have resulted in missing some of the transcripts. We also suggest that the sequencing strategy used for previously published assemblies (which was often a combination of 454 and Illumina technologies), may have produced sub-optimal results. For example, the error-rich 454 sequences may have caused numerous indels in the final contigs, causing difficulties for ORF prediction [45]. Finally, we suggest that the choice of a highly inbred line in our study must have facilitated the *de novo* assembly and produced more continuous and complete contigs (and then, CDSs). Indeed, polymorphism may create complexity in the resolution of the De Bruijn graph resulting in more fragmented results.

Overall, one third of the assembled transcripts showed similarity with known proteins and, in particular 70.8% of the contigs larger than 1 kb were annotated. The majority of the best hits (71.1%) were matches to *Ixodes* tick species, as expected given that a complete genome sequence is available for *I. scapularis* [46]. A smaller fraction had best matches to other groups, primarily arthropods (19.6%). These matches could be genes that are not found in other tick genomic resources due to their relative incompleteness or to the true absence of homologous genes in other tick species. A small number (*n* = 366) of contigs were assigned to genes of mice and rabbit (on which the nymphs and adults had fed), which indicates that host RNA was ingested during the blood meal. As expected, the mean identity at the amino acid level reflected phylogenetic distance, with one exception. We found a higher mean amino acid identity of our transcripts to *I. scapularis* than to *I. ricinus*. One explanation for this counter-intuitive result is that the available genomic resources are more complete for *I. scapularis* than *I. ricinus*. A complete genome has been published for *I. scapularis* [46], but only a genome survey is available for *I. ricinus* [17, 47]. For *I. ricinus*, most protein sequences in the databases are derived from *de novo* assembled transcriptomes that are still rather incomplete. Thus, one explanation for the relatively low mean identity of the hits to *I. ricinus* could be the absence of the same gene in the data banks (matches would often correspond to paralogous gene copies or shared protein domains).

### Gene expression in response to blood-feeding

Differential expression (DE) analyses were focused on the comparison between two feeding conditions, the unfed and partially fed ticks. For this, we had to cope with two potential difficulties, (i) a relatively high rRNA contamination in four of the libraries, and (ii) an imperfect clustering of libraries with regard to sex, stage and feeding condition. These two aspects may be related, since one of the libraries (I, for males) both had a high rRNA content and appeared as a clear outlier, which lead us to discard it from DE analyses. Replicates of a same feeding condition × stage × sex condition are expected to cluster together, and we observed indeed this pattern for unfed nymphs, fed nymphs and unfed adult females. But the two remaining male samples did not cluster together, and there was also an incomplete grouping for the fed females’ libraries. Because it was not clear how to determine which grouping was the most legitimate, we preferred not to censor further libraries. Rather we expect that the inclusion of all “unfed” libraries and all the “fed” libraries together should buffer possible adverse effects of the imperfect clustering, but also lead to sort out the transcripts with the strongest effects in terms of expression change associated with feeding condition (this represents therefore a conservative approach).

DE analysis showed that 11,322 transcripts were either up- or downregulated during blood-feeding. The enrichment tests for unfed and partially fed conditions compared the GO terms between the differentially expressed transcripts and 39,719 unbiased transcripts. The three most important enriched GO terms were related to production of the tick cuticle. Over the course of the blood meal (3 to 8 days depending on the stage), hard ticks undergo dramatic changes in body size. When adult females reach repletion, their body weight increases by a factor of 100 [48]; which means that ticks must completely remodel their cuticle during repletion. Previous studies on adult female *I. ricinus* ticks observed an increase in cuticle thickness from 30 μm to 105 μm during the slow phase (first phase) of engorgement, followed by a decrease to 45 μm during the rapid phase (second phase) of engorgement [20, 43, 48, 49]. These studies support our finding that *I. ricinus* ticks increase their production of cuticular proteins during the blood meal, in order to greatly expand their body size. The next significant GO term (for Molecular Function) associated with upregulated transcripts in the fed condition was GO:0004222 (metalloendopeptidase activity) (Additional file 1: Table S4).

Several studies of the transcriptome of *I. ricinus* during the blood meal have shown the prominent role of several functional groups [12–16, 18, 19], including metalloproteases [12–14] and proteases inhibitors, such as the Kunitz-BPTI group [12, 14, 16]. Unexpectedly, with the exception of the single term found for metalloproteases, these groups of genes were not identified in the GO-enrichment tests made for the comparison between unfed and partially fed ticks. We therefore found only limited overlap between our work and previous studies in terms of GO functions of upregulated genes. We now discuss the possible origins of these differences among our work and previous studies. One reason could be the relatively limited fraction of genes with a GO assignation (15%), possibly resulting in insufficient statistical power in the present work. A second reason is the fact that the previous studies investigated specific tissues (e.g. salivary glands) whereas our study concerned whole ticks. If expression of secreted BP Kunitz proteins is mostly restricted to the tick salivary glands, then any change in gene expression would be diluted when considering the whole tick body where other metabolic processes dominate, such as those related to cuticle production. Our work therefore gives an indication of change of expression only at the very global level of the whole body, and cannot pretend to reach the same level of description of fine-scale changes between tissues, or between precise time-points of the blood meal. A third reason is that GO terms associated with BP Kunitz proteins, metalloproteases, reprolysin, etc., were not exclusively upregulated in the partially fed ticks. In fact, several of the genes that were considered as unbiased or genes that were strongly upregulated in unfed ticks had the same GO terms. The same observation applies to other terms that have been associated with blood-feeding in previous studies, or to other terms (e.g. zinc finger). The text mining analysis of the PFAM domains also found a high frequency of terms like reprolysin and Kunitz domains in the upregulated transcripts of the partially fed ticks. This result is in agreement with previous publications [12–16, 18, 19]. However, we note that these domains are also common in the upregulated transcripts in unfed ticks. This observation suggests that ticks contain multigenic families that share these common domains and that different genes in these families are expressed at different points in the tick life-cycle. This observation also suggests a subtle transcriptional landscape, where shifts in gene expression should be studied at the level of the gene rather than at the level of blocks of genes sharing the same GO or domain assignation.

### Genetic diversity

We therefore established a catalogue of robust SNPs which could be useful for future population genetics analyses, as it could be used to design a genotyping study of multiple individuals, allowing a shift in scale compared to traditional population genetics approaches based on small numbers of markers. To our knowledge, only one similar analysis with RNAseq data has been conducted to date for *I. ricinus* [14], but the numbers of identified SNPs between this study and our work are difficult to compare given the different strategies (*de novo* SNP identification from the reads *versus* mapping to a set of CDSs) and because the reference sets used for localization of the SNPs are not the same. We however stress that the *de novo* (direct from the reads) approach for RNAseq datasets has been successfully applied in recent studies for different organisms [50]. Even though the different datasets analysed in this work comprise pools of individuals or materials of very different origins, our approach illustrates the power of these analyses to determine the genetic characteristics of the different materials. Our study of single nucleotide polymorphisms found indeed substantial differences in heterozygosity between the different sources of *I. ricinus* used in RNAseq studies. The factorial component analysis indicated that three datasets corresponding to wild ticks (SEN, CZ-W and LUX) clustered together, whereas each of the three laboratory strains (NEU, CZ-F1 and CL) differed markedly from that cluster and from each other. The clustering of the wild tick populations indicates that they have similar levels of heterozygosity (and should not be interpreted as the absence of genetic diversity between the three wild tick populations). In contrast, the laboratory lines show reduced levels of heterozygosity and frequent allele fixation at each of the SNP sites (Additional file 1: Figure S7). Reduction in heterozygosity was expected in the CZ-F1 strain because it was derived from a single mating of two wild ticks. Reduced heterozygosity was also expected in the NEU strain because it was derived from a laboratory colony that has a long history of inbreeding and small effective population size. The material from the tick cell line (CL) had an even more extreme reduction of heterozygosity, which is a typical feature of cell lines [51]. The laboratory strains differ from each other because of genetic drift, which results in the random fixation of alleles in each laboratory strain. Our study illustrates that sequencing the transcriptome of tick populations allows the genotyping of thousands of SNPs at hundreds of genes, which can greatly extend the traditional populations genetic approaches based on much fewer genetic markers [14, 42, 50].

## Conclusions

Our *de novo* assembly showed high overall completeness and significantly enlarged the catalogue of known coding sequences for *I. ricinus*. Our study investigated the transcriptome of whole ticks that differed with respect to their developmental stage, sex and feeding condition. This approach allowed us to detect changes in gene expression at the level of the whole body instead of specific tissues. We found that blood-feeding induced a strong upregulation of transcripts associated with cuticle production. Finally, our analysis of polymorphism in the transcriptome sequence data of our study and previously published studies allowed us to identify 3866 robust SNPs from expressed-regions, and to compare the levels of genetic diversity between outbred wild strains of *I. ricinus versus* inbred laboratory strains and tick cell lines.

## Additional file

Additional file 1: **Table S1.** Annotation of a rRNA containing contig from a preliminary *de novo* assembly. **Table S2.** Assembly statistics for Transcriptome Shotgun Assembly datasets (*de novo* assemblies corresponding to published papers, or still unpublished). **Table S3.** Details of published RNAseq studies for *I. ricinus*. **Table S4.** GO Enrichment for partially fed ticks (Molecular Function). **Table S5.** GO Enrichment for partially fed ticks (Biological Process). **Table S6.** GO Enrichment for partially fed ticks (Cellular Component). **Figure S1.** Reads sub-sampling and assembly statistics. **Figure S2.** Reads sub-sampling and assembly completeness. **Figure S3.** Quantification of Fungi-like reads in the different libraries. **Figure S4.** Distribution of the contig’s identity with Acari and *Ixodes* species. **Figure S5.** Expression of the 200 most expressed genes among all libraries. **Figure S6.** Heatmap showing the hierarchical clustering of the 15 libraries based on expression counts. **Figure S7.** Distribution of estimated heterozygosity for six populations, using SNPs discovered by KisSplice. (PDF 380 kb)

## Abbreviations

BP: Biological Process
CC: Cellular Component
DE: Differential Expression
FCA: Factorial Component Analysis
GO: Gene Ontology
MAF: Minor Allele Frequency
MF: Molecular Function
ORFs: Open Reading Frame
SNPs: Single Nucleotide Polymorphisms
SRA: Sequence Read Archive
T-DBG: Transcriptome De Bruijn Graph
TSA: Transcriptome Sequence Assembly

## References

[1] Jongejan F, Uilenberg G. The global importance of ticks. Parasitology. 2004;129(Suppl.):S3–S14. 10.1017/s0031182004005967 15938502

[2] de la Fuente J, Antunes S, Bonnet S, Cabezas-Cruz A, Domingos AG, Estrada-Peña A (2017). Tick-pathogen interactions and vector competence: identification of molecular drivers for tick-borne diseases. Front Cell Infect Microbiol..

[3] Kazimírová M, Štibrániová I (2013). Tick salivary compounds: their role in modulation of host defences and pathogen transmission. Front Cell Infect Microbiol..

[4] Brossard M, Wikel S (2004). Tick immunobiology. Parasitology..

[5] Hovius JW, van Dam AP, Fikrig E (2007). Tick-host-pathogen interactions in Lyme borreliosis. Trends Parasitol..

[6] Ramamoorthi N, Narasimhan S, Pal U, Bao F, Yang XF, Fish D (2005). The Lyme disease agent exploits a tick protein to infect the mammalian host. Nature..

[7] Kung F, Anguita J, Pal U (2013). *Borrelia burgdorferi* and tick proteins supporting pathogen persistence in the vector. Future Microbiol..

[8] Schuijt TJ, Hovius JW, van der Poll T, van Dam AP, Fikrig E (2011). Lyme borreliosis vaccination: the facts, the challenge, the future. Trends Parasitol..

[9] Rizzoli A, Hauffe HC, Carpi G, Vourc’h GI, Neteler M, Rosa R (2011). Lyme borreliosis in Europe. Eurosurveillance..

[10] Süss J (2011). Tick-borne encephalitis 2010: epidemiology, risk areas, and virus strains in Europe and Asia - an overview. Ticks Tick Borne Dis..

[11] Milano I, Babbucci M, Panitz F, Ogden R, Nielsen RO, Taylor MI (2011). Novel tools for conservation genomics: comparing two high-throughput approaches for SNP discovery in the transcriptome of the European hake. PloS One..

[12] Chmelař J, Anderson JM, Mu J, Jochim RC, Valenzuela JG, Kopecký J (2008). Insight into the sialome of the castor bean tick, *Ixodes ricinus*. BMC Genomics..

[13] Schwarz A, von Reumont BM, Erhart J, Chagas AC, Ribeiro JM, Kotsyfakis M (2013). *De novo Ixodes ricinus* salivary gland transcriptome analysis using two next-generation sequencing methodologies. FASEB J..

[14] Kotsyfakis M, Schwarz A, Erhart J, Ribeiro JM (2015). Tissue-and time-dependent transcription in *Ixodes ricinus* salivary glands and midguts when blood-feeding on the vertebrate host. Sci Rep..

[15] Kotsyfakis M, Kopáček P, Franta Z, Pedra JHF, Ribeiro JMC (2015). Deep sequencing analysis of the *Ixodes ricinus* haemocytome. PLOS Negl Trop Dis..

[16] Liu XY, de la Fuente J, Cote M, Galindo RC, Moutailler S, Vayssier-Taussat M (2014). IrSPI, a tick serine protease inhibitor involved in tick feeding and *Bartonella henselae* infection. PLoS Negl Trop Dis..

[17] Cramaro WJ, Revets D, Hunewald OE, Sinner R, Reye AL, Muller CP (2015). Integration of *Ixodes ricinus* genome sequencing with transcriptome and proteome annotation of the naïve midgut. BMC Genomics..

[18] Schwarz A, Tenzer S, Hackenberg M, Erhart J, Gerhold-Ay A, Mazur J (2014). A systems level analysis reveals transcriptomic and proteomic complexity in *Ixodes ricinus* midgut and salivary glands during early attachment and feeding. Mol Cell Prot..

[19] Perner J, Provazník J, Schrenková J, Urbanová V, Ribeiro JM, Kopáček P (2016). RNA-seq analyses of the midgut from blood-and serum-fed *Ixodes ricinus* ticks. Sci Rep..

[20] Lees A (1952). The role of cuticle growth in the feeding process of ticks. Proc Zool Soc London..

[21] Bolger AM, Lohse M, Usadel B (2014). Trimmomatic: a flexible trimmer for Illumina sequence data. Bioinformatics..

[22] Andrews S. FastQC: A quality control tool for high throughput sequence data. http://www.bioinformatics.babraham.ac.uk/projects/fastqc/. Accessed 10 Oct 2015.

[23] Langmead B, Salzberg SL (2012). Fast gapped-read alignment with Bowtie 2. Nat Methods..

[24] Haas BJ, Papanicolaou A, Yassour M, Grabherr M, Blood PD, Bowden J (2013). *De novo* transcript sequence reconstruction from RNA-seq using the Trinity platform for reference generation and analysis. Nat Protoc..

[25] Simão FA, Waterhouse RM, Ioannidis P, Kriventseva EV, Zdobnov EM (2015). BUSCO: assessing genome assembly and annotation completeness with single-copy orthologs. Bioinformatics..

[26] Camacho C, Coulouris G, Avagyan V, Ma N, Papadopoulos J, Bealer K, Madden TL (2009). BLAST+: architecture and applications. BMC Bioinformatics..

[27] Eddy SR (2011). Accelerated profile HMM searches. PLoS Comput Biol..

[28] Li W, Godzik A (2006). Cd-hit: a fast program for clustering and comparing large sets of protein or nucleotide sequences. Bioinformatics..

[29] Trinotate: Transcriptome Functional Annotation and Analysis. https://trinotate.github.io/. Accessed 1 Feb 2016

[30] Finn RD, Bateman A, Clements J, Coggill P, Eberhardt RY, Eddy SR (2013). Pfam: the protein families database. Nucleic Acids Res..

[31] Petersen TN, Brunak S, Von Heijne G, Nielsen H (2011). SignalP 4.0: discriminating signal peptides from transmembrane regions. Nat Methods..

[32] Krogh A, Larsson B, Von Heijne G, Sonnhammer EL (2001). Predicting transmembrane protein topology with a hidden Markov model: application to complete genomes. J Mol Biol..

[33] Lagesen K, Hallin P, Rødland EA, Stærfeldt HH, Rognes T, Ussery DW (2007). RNAmmer: consistent and rapid annotation of ribosomal RNA genes. Nucleic Acids Res..

[34] Ioannidou ZS, Theodoropoulou MC, Papandreou NC, Willis JH, Hamodrakas SJ (2014). CutProtFam-Pred: detection and classification of putative structural cuticular proteins from sequence alone, based on profile hidden Markov models. Insect Biochem Mol Biol..

[35] Bray NL, Pimentel H, Melsted P, Pachter L (2016). Near-optimal probabilistic RNA-seq quantification. Nat Biotechnol..

[36] Love MI, Huber W, Anders S (2014). Moderated estimation of fold change and dispersion for RNA-seq data with DESeq2. Genome Biol..

[37] Harris MA, Clark J, Ireland A, Lomax J, Ashburner M, Foulger R (2004). The Gene Ontology (GO) database and informatics resource. Nucleic Acids Res..

[38] Alexa A, Rahnenfuhrer J. topGO: Enrichment Analysis for Gene Ontology. R package version 2.18.0: 2010. http://master.bioconductor.org/packages/devel/bioc/citations/topGO/citation.html.

[39] Sanchez A, Ocana J, Salicru M. goProfiles: an R Package for the Statistical Analysis of Functional Profiles. R package version 1.28.0; 2010.

[40] Sacomoto GA, Kielbassa J, Chikhi R, Uricaru R, Antoniou P, Sagot MF (2012). KISSPLICE: de-novo calling alternative splicing events from RNA-seq data. BMC Bioinformatics..

[41] Kent WJ (2002). BLAT - the BLAST-like alignment tool. Genome Res..

[42] Lopez-Maestre H, Brinza L, Marchet C, Kielbassa J, Bastien S, Boutigny M (2016). SNP calling from RNA-seq data without a reference genome: identification, quantification, differential analysis and impact on the protein sequence. Nucleic Acids Res..

[43] Andersen SO, Roepstorff P (2005). The extensible alloscutal cuticle of the tick, *Ixodes ricinus*. Insect Biochem Mol Biol.

[44] Kim TK, Tirloni L, Pinto AFM, Moresco J, Yates JRIII, da Silva Vaz I (2016). *Ixodes scapularis* tick saliva proteins sequentially secreted every 24 h during blood-feeding. PLOS Negl Trop Dis..

[45] Francis WR, Christianson LM, Kiko R, Powers ML, Shaner NC, Haddock SH. A comparison across non-model animals suggests an optimal sequencing depth for *de novo* transcriptome assembly. BMC Genomics. 2013;14:167. 10.1186/1471-2164-14-167 PMC3655071 23496952

[46] Gulia-Nuss M, Nuss AB, Meyer JM, Sonenshine DE, Roe RM, Waterhouse RM (2016). Genomic insights into the *Ixodes scapularis* tick vector of Lyme disease. Nat Commun..

[47] Cramaro WJ, Hunewald OE, Sakyi-Lesley L, Muller CP (2017). Genome scaffolding and annotation for the pathogen vector *Ixodes ricinus* by ultra-long single molecule sequencing. Parasit Vectors..

[48] Flynn PC, Kaufman WR (2011). Female ixodid ticks grow endocuticle during the rapid phase of engorgement. Exp Appli Acarol..

[49] Dillinger S, Kesel A (2002). Changes in the structure of the cuticle of *Ixodes ricinus* L., 1758 (Acari, Ixodidae) during feeding. Arthropod Struct Dev..

[50] De Wit P, Pespeni MH, Palumbi SR (2015). SNP genotyping and population genomics from expressed sequences - current advances and future possibilities. Mol Ecol..

[51] Tischfield JA (1997). Loss of heterozygosity or: how I learned to stop worrying and love mitotic recombination. Am J Hum Genet..

