# Supplementary material for: Whole body transcriptomes and new insights into the biology of the tick Ixodes ricinus
Source: Parasit Vectors. 2018 Jun 26;11:364. doi: 10.1186/s13071-018-2932-3 (PMC6019515; doi:10.1186/s13071-018-2932-3)
Supplement: Supplementary file 1 — Table S1. Annotation of a rRNA containing contig from a preliminary de novo assembly. Table S2. Assembly statistics for Transcriptome Shotgun Assembly datasets (de novo assemblies corresponding to published papers, or still unpublished). Table S3. Details of published RNAseq studies for I. ricinus. Table S4. GO Enrichment for partially fed ticks (Molecular Function). Table S5. GO Enrichment for partially fed ticks (Biological Process). Table S6. GO Enrichment for partially fed ticks (Cellular Component). Figure S1. Reads sub-sampling and assembly statistics. Figure S2. Reads sub-sampling and assembly completeness. Figure S3. Quantification of Fungi-like reads in the different libraries. Figure S4. Distribution of the contig’s identity with Acari and Ixodes species. Figure S5. Expression of the 200 most expressed genes among all libraries. Figure S6. Heatmap showing the hierarchical clustering of the 15 libraries based on expression counts. Figure S7. Distribution of estimated heterozygosity for six populations, using SNPs discovered by KisSplice. (PDF 380 kb) [file 13071_2018_2932_MOESM1_ESM.pdf]

Supplemental material of “Whole body transcriptomes and new insights into the biology of the tick *Ixodes ricinus*”

N. Pierre Charrier[1], Marjorie Couton[1], Maarten J. Voordouw[2], Olivier Rais[2], Axelle Durand-Hermouet[1], Caroline Hervet[1], Olivier Plantard[1] and Claude Rispe[1]

[1]BIOEPAR, INRA, Oniris, Université Bretagne Loire, 44307, Nantes, France
[2]Laboratoire d’Ecologie et Evolution des parasites, Institut de Biologie, Université de Neuchâtel, Rue Emile-Argand 11, CH-2000, Neuchâtel, Switzerland

May 29, 2018

List of Figures

S1 Reads sub-sampling and assembly statistics 5
S2 Reads sub-sampling and assembly completeness 6
S3 Quantification of Fungi-like reads in the different libraries 7
S4 Distribution of the contig’s identity with Acari and Ixodes species 8
S5 Expression of the 200 most expressed genes among all libraries 9
S6 Hierarchical clustering of the 15 libraries 10
S7 Heterozygosity distribution for six populations 11

List of Tables

S1 Annotation of the rRNA contig 2
S2 Assembly statistics for Transcriptome Shotgun Assembly data sets 3
S3 Details of published RNAseq studies for *I. ricinus* 4
S4 GO Enrichment for partially fed ticks (Molecular Function) 12
S5 GO Enrichment for partially fed ticks (Biological Process) 12
S6 GO Enrichment for partially fed ticks (Cellular Component) 13

Table S1: Annotation of a rRNA containing contig from a preliminary *de novo* assembly. Annotation of this contig with Rfam (release 12.1 2016-04-26) confirmed the presence of three rRNA units 18S (SSU: positions 3102-7008), 5.8S (positions 2244-2396) and 28S (LSU: positions 3102-7008).

ID	accession	start	end	bits score	E-value	strand
LSU_rRNA_eukarya	RF02543	3102	7008	2943.5	0	+
SSU_rRNA_eukarya	RF01960	1	1816	1773.3	3.7e-34	+
SSU_rRNA_bacteria	RF00177	1	1821	359.3	3.1e-13	+
SSU_rRNA_microsporidia	RF02542	1	1816	874.4	5e-266	+
5_8S_rRNA	RF00002	2244	2396	126.9	1.5e-32	+
SSU_rRNA_archaea	RF01959	1	1819	437.0	7.5e-14	+
LSU_rRNA_bacteria	RF02541	2991	6751	1056.8	0	+
LSU_rRNA_archaea	RF02540	3032	6757	1271.4	0	+

Table S2: Assembly statistics for Transcriptome Shotgun Assembly data sets (*de novo* assemblies corresponding to published papers, or still unpublished).

Name	GADI	GANP	GBIH	GCJO	GEFM	GEGO
BioProject	PRJNA177622	PRJNA217984	PRJNA183509	PRJNA270980	PRJNA311553	PRJNA312361
Date	July 2015	March 2015	September 2014	April 2015	February 2016	April 2016
Study	Schwarz, 2013[13]	Schwarz, 2014[18]; Kotsyfakis, 2015[14]	Kotsyfakis, 2015[15]	Cramaro, 2015[17]	Perner, 2016[19]	Unpublished
Number of CDS	8,685	16,002	2,854	(25,962)[1]	7,215	7,692
Number of contigs	8,685	16,002	2,854	59,924	7,215	7,692
total size of contigs	5,465,358	14,483,137	2,128,460	25,311,446	9,540,547	9,280,828
Shortest contig	201	201	201	200	201	201
Longest contig	5,352	16,869	6,909	9,464	17,475	19,044
Number of contigs $>$ 500	4,343	10,145	1,599	12,771	5,933	5,231
Number of contigs $>$ 1k	1,269	4,668	523	2,664	3,724	3,317
Number of contig $>$ 10k	0	7	0	0	10	6
mean contig size	629	905	746	422	1,322	1,207
median contig size	501	645	549	333	1,026	846
N50 contig length	741	1,212	882	437	1,683	1,818

1 - GCJO consists in a collection of contigs, number in perenthesis is the number of CDSs predicted by TransDecoder.
References are numbered accordingly to the list of cited references in the main manuscript.

Table S3: Details of published RNAseq studies for *I. ricinus*. First column, data set acronym (Strain). Second column, type of material: wild ticks, F1 between wild ticks, Cell line, (Origin). Third column, NCBI BioProject accession number. Column 4, accession number (TSA). Column 5, references. Column 6, sequencing technology used. Column 7, total amount of bases sequenced in Gigabases. Column 8, tissues used (SG, salivary glands, H, haemocytome, MG, midgut). Column 9, stages (Ny, nymphs, Ad, adults). Column 10, condition (U, unfed, Pfed, for partially fed ,F, fully fed).

Strain	Origin	BioProject	TSA	Study	Technology	Gb.	Tissues	Stage	Condition
CZ-W	Wild	PRJNA183509	GBIH	Kotsyfakis, 2015 [15]	Illumina	29.9	SG, H	Ny, Ad	Pfed and F
		PRJNA177622	GADI	Schwarz, 2013 [13]	Illumina, 454	8.87	SG, H	Ny, Ad	Pfed and F
CZ-F1	F1	PRJNA312361	GEGO	Unpublished	Illumina	24	SG	Fe	N.A.
		PRJNA311553	GEFM	Perner, 2016 [19]	Illumina	56	MG	Fe	Pfed and F
LUX	Wild	PRJNA270980	GCJO	Cramaro, 2015[17]	Ion torrent	4.3	MG	Ad	U
CL	Cell line	PRJNA238785			Illumina	1.9	CL	-	-
		PRJNA238786			Illumina	1.5	CL	-	-
		PRJNA238787			Illumina	2.4	CL	-	-
		PRJNA238788			Illumina	2.7	CL	-	-
SEN	Wild	PRJNA237360		Liu, 2014 [16]	Illumina	17.7	SG	Ad	Pfed

References are numbered accordingly to the list of cited references in the main manuscript.

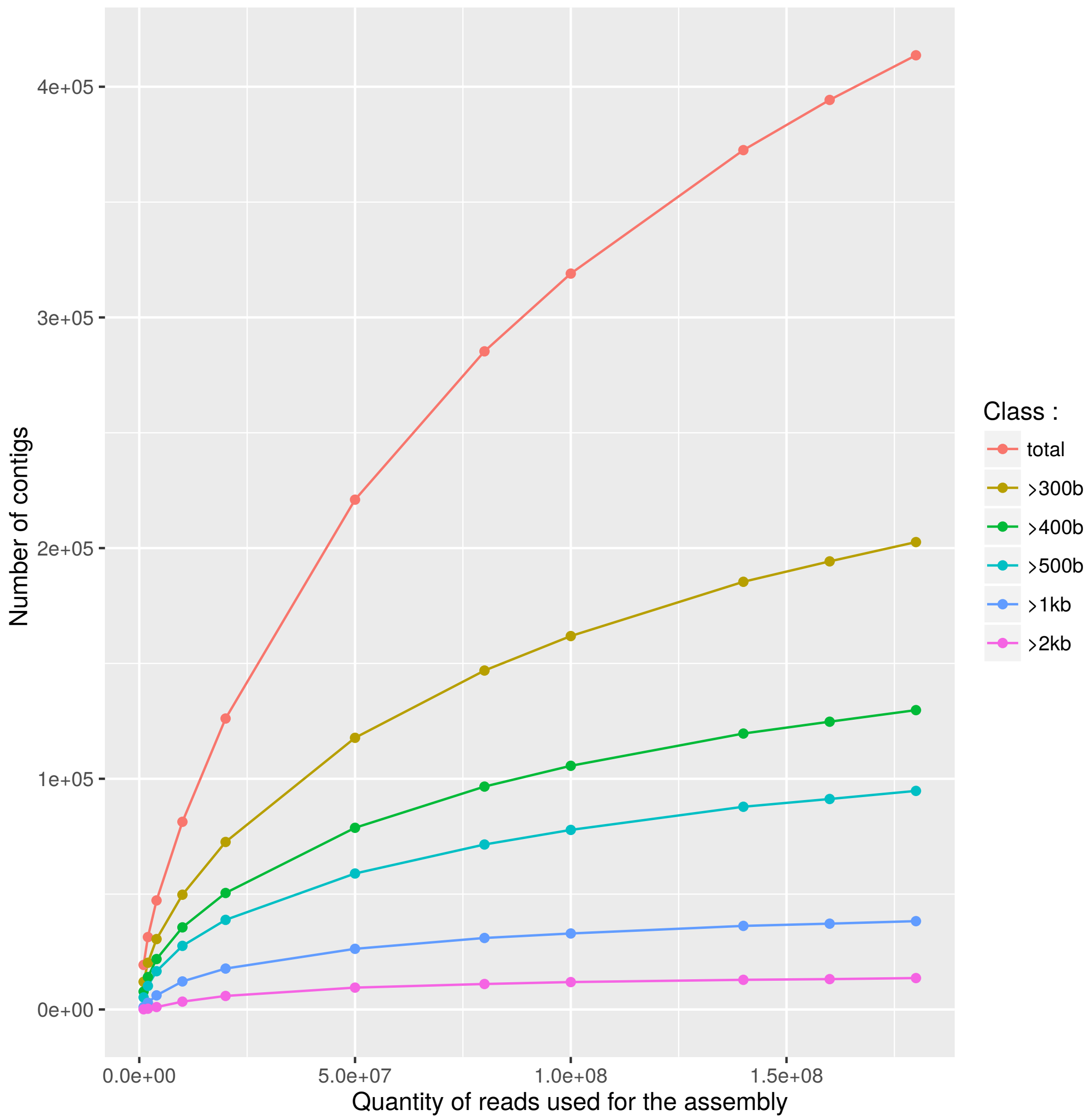

Figure S1: Reads sub-sampling and assembly statistics. Number of contigs obtained after a *de novo* assembly with `Trinity`, for different sample size (number of reads in abscissus). Numbers of transcripts: total (red), transcripts $>$ 300bp (yellow), $>$ 400bp (green), $>$ 500bp (light blue), >1kb (marine blue), and >2kb (purple).

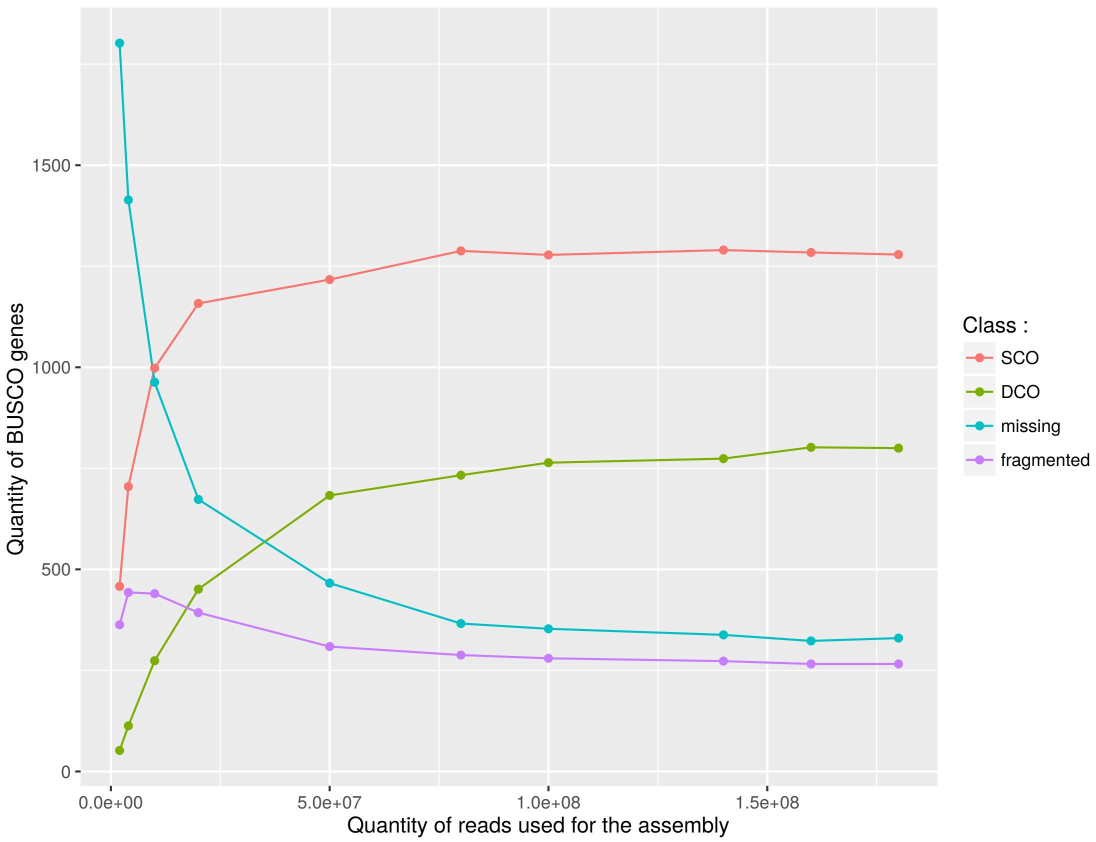

Figure S2: Reads sub-sampling and assembly completeness. Number of BUSCO genes (y-axis) found as a function of sample sizes of reads used for the assembly (number of reads sampled in abscissus). Abbreviations: SCO, Single Copy Orthoguous BUSCO genes; DCO, Duplicated Copy Orthologs; missing, number of absent BUSCO genes; fragmented: BUSCO genes found partially.

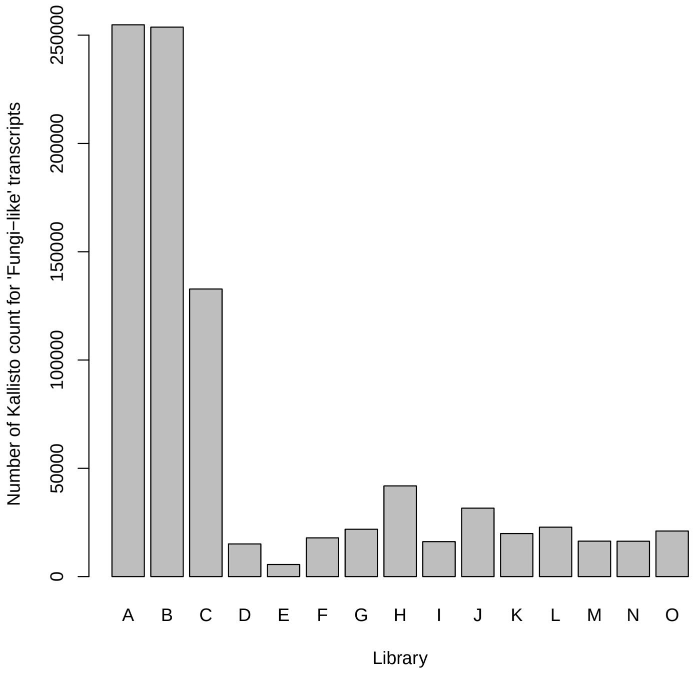

Figure S3: Quantification of Fungi-like reads in the different libraries. Total of expression counts (with Kallisto) for all contigs showing similarity with Fungi, based on Uniref90 annotation (first hit to Fungi, proteic identity $> 50\%$ and E-value lower than $10e-5$). The total of expression counts across all the libraries for Fungi-like transcripts was 887,767, for total of 72,392,431 `kallisto` counts (1.23%).

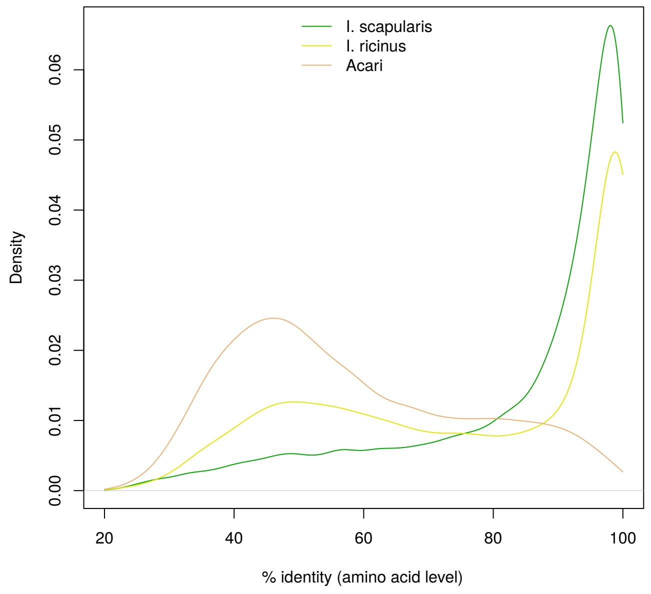

Figure S4: Distribution of the contig's identity with Acari and Ixodes species. Distribution of the best hit identity (at the amino acid level) on Uniref90 for two *Ixodes* species and the "Acari" taxon (other species of Acari).

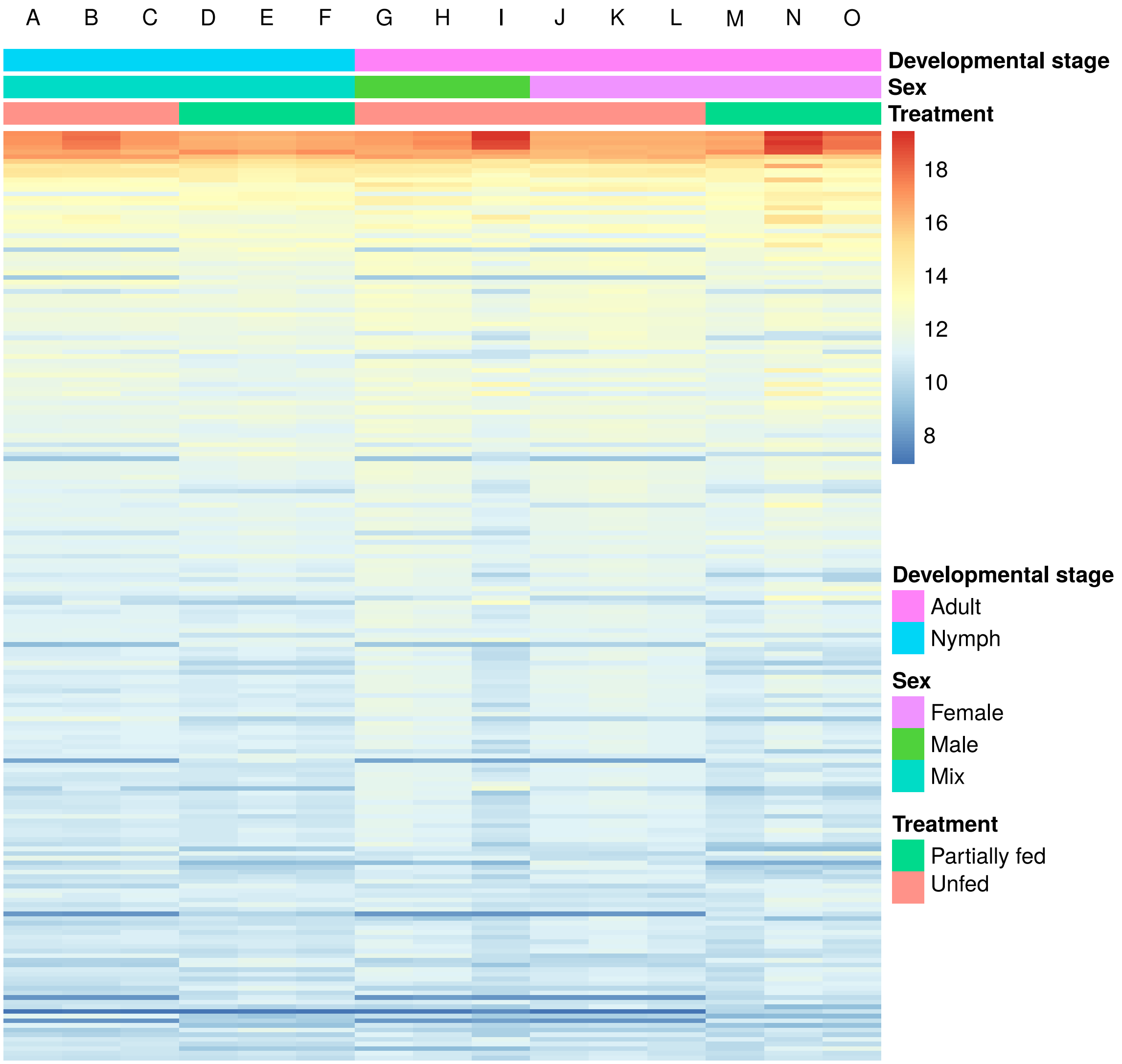

Figure S5: Expression of the 200 most expressed genes among all libraries represented by a color scale (red for highest expression, blue for lowest expression) and measured by a log2-transformed Kallisto count.

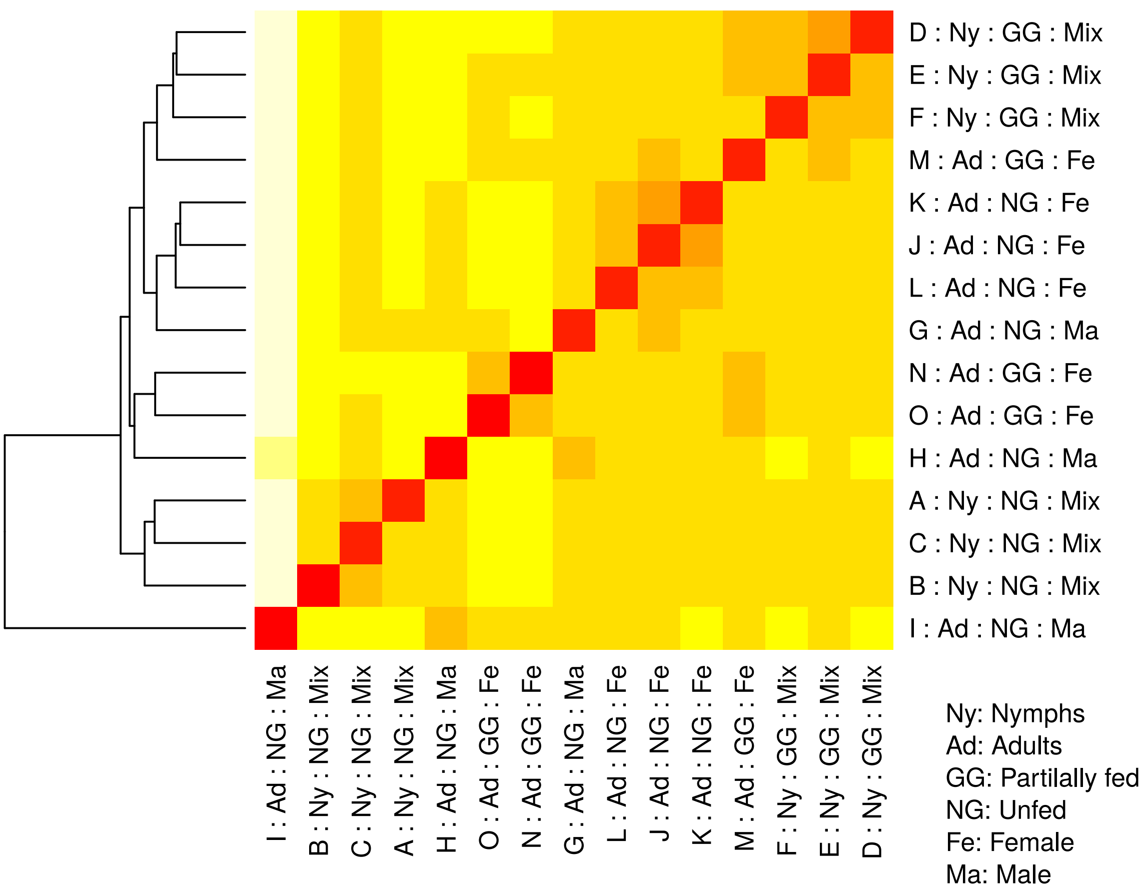

Figure S6: Heatmap showing the hierarchical clustering of the 15 libraries based on expression counts.

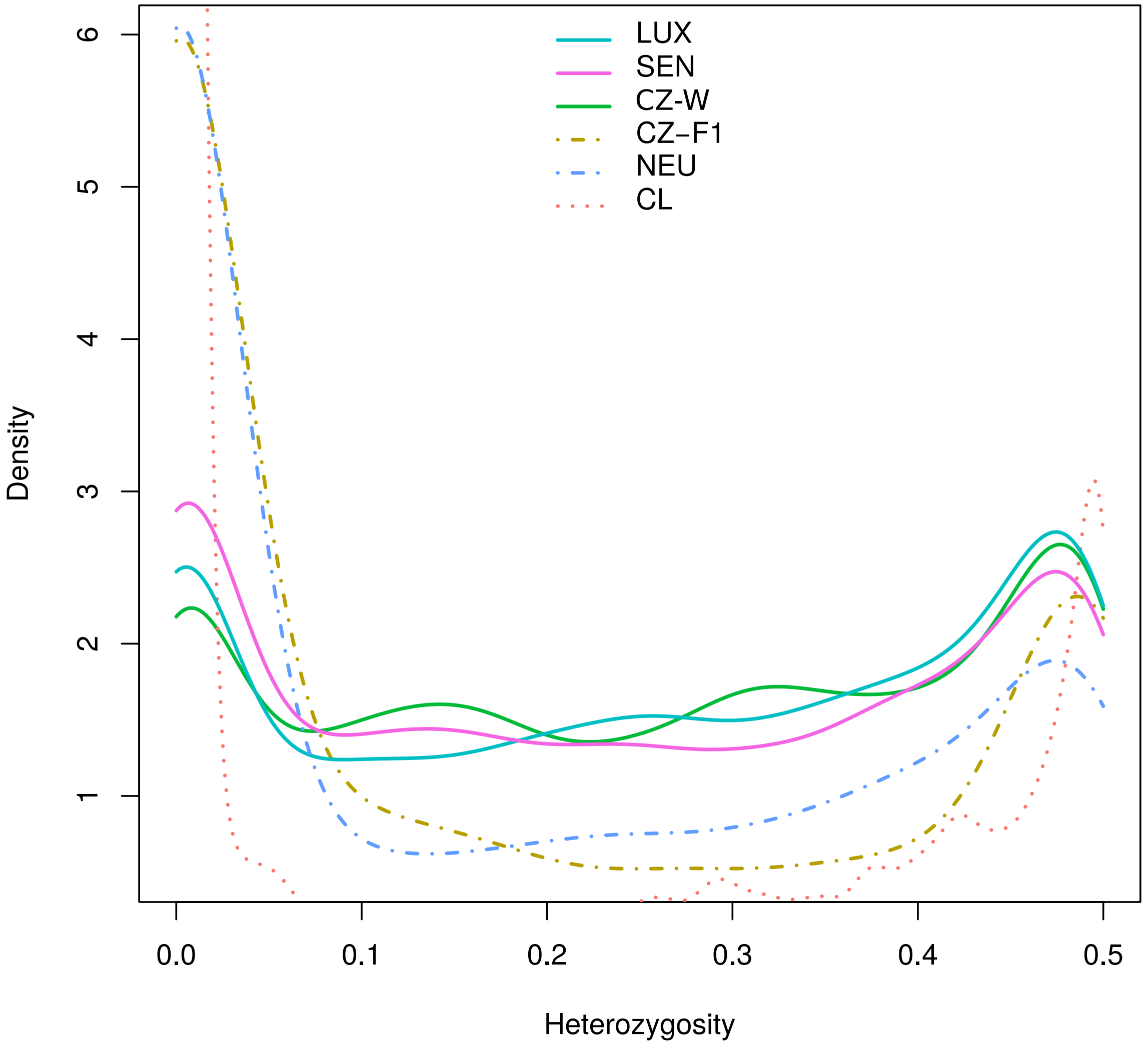

Figure S7: Distribution of estimated heterozygosity for six populations, using SNPs discovered by `KisSplice`. Heterozygosity was calculated on well-covered loci for each of the six data set (n=3,866), estimating variant frequency ($He = 2pq$) for each locus and each data set.

Table S4: GO Enrichment for partially fed ticks (Molecular Function). The elimKS column represents the significativity of the the Elim-Kolmogorov Smirnoff test implemented in the topGO R package, maintained by Adrian Alexa and Jorg Rahnenfuhrer. This tests for enriched GO terms in genes significantly over-expressed in partially fed ticks in comparison with unbiased genes.

GO.ID	Term	elimKS	adjustedFDR
GO:0008061	chitin binding	1.8e-10	9.0e-09
GO:0042302	structural constituent of cuticle	2.6e-06	6.5e-05
GO:0005201	extracellular matrix structural constituent	0.0011	0.018
GO:0004222	metalloendopeptidase activity	0.0021	0.026

Table S5: GO Enrichment for partially fed ticks (Biological Process). The elimKS column represents the significativity of the the Elim-Kolmogorov Smirnoff test implemented in the topGO R package, maintained by Adrian Alexa and Jorg Rahnenfuhrer. This tests for enriched GO terms in genes significantly over-expressed in partially fed ticks in comparison with unbiased genes.

GO.ID	Term	elimKS	adjustedFDR
GO:0006030	chitin metabolic process	1.1e-05	0.00055
GO:0006457	protein folding	0.0019	0.0432
GO:0006508	proteolysis	0.0032	0.0432
GO:0071214	cellular response to abiotic stimulus	0.0045	0.0432
GO:0048608	reproductive structure development	0.0059	0.0432
GO:0040014	regulation of multicellular organism growth	0.0071	0.0432
GO:0006729	tetrahydrobiopterin biosynthetic process	0.0077	0.0432
GO:0006091	generation of precursor metabolites and energy	0.0106	0.0432
GO:0072593	reactive oxygen species metabolic process	0.0111	0.0432
GO:0045333	cellular respiration	0.0111	0.0432
GO:0030707	ovarian follicle cell development	0.0113	0.0432
GO:0009056	catabolic process	0.0126	0.0432
GO:0044248	cellular catabolic process	0.0127	0.0432
GO:0072001	renal system development	0.0139	0.0432
GO:0002064	epithelial cell development	0.0165	0.0432

GO:0031669	cellular response to nutrient levels	0.0184	0.0432
GO:0001666	response to hypoxia	0.0201	0.0432
GO:0036293	response to decreased oxygen levels	0.0201	0.0432
GO:0070482	response to oxygen levels	0.0201	0.0432
GO:0001655	urogenital system development	0.0206	0.0432
GO:0043648	dicarboxylic acid metabolic process	0.0207	0.0432
GO:0016192	vesicle-mediated transport	0.0211	0.0432
GO:0015980	energy derivation by oxidation of organic compounds	0.0214	0.0432
GO:0002065	columnar/cuboidal epithelial cell differentiation	0.0216	0.0432
GO:0002066	columnar/cuboidal epithelial cell cell development	0.0216	0.0432
GO:0051641	cellular localization	0.0231	0.0444
GO:0042743	hydrogen peroxide metabolic process	0.0277	0.0478
GO:0042744	hydrogen peroxide catabolic process	0.0277	0.0478
GO:0019220	regulation of phosphate metabolic process	0.0287	0.0478
GO:0051174	regulation of phosphorus metabolic process	0.0287	0.0478
GO:0061326	renal tubule development	0.0338	0.0491
GO:0061333	renal tubule morphogenesis	0.0338	0.0491
GO:0006099	tricarboxylic acid cycle	0.0355	0.0491
GO:0008406	gonad development	0.0368	0.0491
GO:0045137	development of primary sexual characteristics	0.0368	0.0491
GO:0000281	mitotic cytokinesis	0.0382	0.0491
GO:0061640	cytoskeleton-dependent cytokinesis	0.0382	0.0491
GO:0044743	intracellular protein transmembrane import into intracellular organelle	0.0399	0.0491
GO:0065002	intracellular protein transmembrane transport	0.0399	0.0491
GO:0071806	protein transmembrane transport	0.0399	0.0491
GO:0006536	glutamate metabolic process	0.0403	0.0491

Table S6: GO Enrichment for partially fed ticks (Cellular Component).The elimKS column represents the significativity of the the Elim-Kolmogorov Smirnoff test implemented in the topGO R package, maintained by Adrian Alexa and Jorg Rahnenfuhrer. This tests for enriched GO terms in genes significantly over-expressed in partially fed ticks in comparison with unbiased genes.

GO.ID	Term	elimKS	adjustedFDR

GO:0005578	proteinaceous extracellular matrix	2.6e-05	0.0013
GO:0005576	extracellular region	0.00031	0.0077
GO:0042470	melanosome	0.00279	0.0331
GO:0005788	endoplasmic reticulum lumen	0.00281	0.0331
GO:0005581	collagen trimer	0.00331	0.0331
GO:0044421	extracellular region part	0.00483	0.0402
